# Phylogenomics, ecomorphological evolution, and historical biogeography in *Deuterocohnia* (Bromeliaceae: Pitcairnioideae)

**DOI:** 10.1002/ajb2.70153

**Published:** 2026-01-28

**Authors:** Bing Li, Nicole Schütz, Kurt Weising, Georg Zizka, Jacob B. Landis, Thomas J. Givnish

**Affiliations:** ^1^ Department of Botany University of Wisconsin‐Madison Madison 53706 WI USA; ^2^ Department of Botany University of Kassel Kassel 34132 Germany; ^3^ Institute of Ecology, Evolution, and Diversity Goethe University and Senckenberg Research Institute Frankfurt am Main 60438 Germany; ^4^ School of Integrative Plant Science and L. H. Bailey Hortorium Cornell University Ithaca 14853 USA NY

**Keywords:** Bromeliaceae, hybridization, Neotropics, next generation sequencing, phylogenetics, target capture

## Abstract

**Premise:**

Species of *Deuterocohnia* (17 spp.) show extraordinary variation in elevation (0–3900 m a.s.l.) and growth forms, and many have narrow geographic distributions in the west‐central Andes and the Peru‐Chile coast. Previous research using few plastid and nuclear loci failed to produce well‐resolved or supported phylogenies. Here we sequenced 1815 single‐copy nuclear genes and whole plastomes to infer relationships, screen for reticulation, reconstruct evolution of vegetative and floral characters, and evaluate species groups and their historical biogeography.

**Methods:**

We developed the Bromeliad1815 bait set to capture low‐copy nuclear genes across Bromeliaceae, producing nuclear and plastome phylogenies for *Deuterocohnia* and outgroups in six bromeliad subfamilies using maximum likelihood, ASTRAL, and network analyses; test for cytonuclear conflict and its potential causes; and evaluate evolution of morphological characters in relation to each other and elevation using phylogenetic PCA and phylogenetic regression.

**Results:**

We produced fully resolved, strongly supported nuclear and plastome phylogenies for *Deuterocohnia*, with crown ages of 5.5 and 8.0 Mya, respectively. Cytonuclear conflict appears driven mainly by hybridization/introgression, consistent with several species co‐occurring in small areas. Vegetative organs and growth form become increasingly compact with elevation, reflecting adaptation to desiccation, wind exposure, and cold soils. *Deuterocohnia* arose in southeastern Bolivia and repeatedly evolved up‐ and downslope into other habitats from Andean Yungas at mid‐elevation.

**Conclusions:**

Our results imply rapid adaptive divergence (e.g., in *strobilifera‐chrysantha*), convergent evolution (two origins of the cushion growth‐form), phylogeny consistent with form in some cases (e.g., *seramisiana‐brevispicata‐meziana*) and recurrent effects of the Rio Pilcomayo barrier on speciation and chloroplast capture.

The monocot family Bromeliaceae—with 80 genera and 3770 species—is the largest of the 52 angiosperm families restricted or nearly so to the Neotropics (Ulloa Ulloa et al., 2017; Gouda and Butcher, 2024) and contains more species of epiphytes (ca. 1800) than any other family except Orchidaceae (Zotz, 2013). Bromeliaceae has radiated extensively in vegetative form, with several key innovations (e.g., absorptive leaf trichomes, tank habit, CAM photosynthesis, epiphytism) that add substantial functional diversity and allow bromeliads to compete successfully across a wide range of ecological conditions (Benzing, 2000; Givnish et al., 1997, 2011, 2014). Historically, Bromeliaceae was split into three subfamilies (Bromelioideae, Pitcairnioideae, Tillandsioideae) distinguished by fruit and seed characteristics (Mez 1934; Smith and Downs, 1974). However, analyses of sequence data for one to eight plastid loci showed that Pitcairnioideae as originally defined by capsular fruits and winged seeds is paraphyletic. To preserve monophyly, Givnish et al. (2007, 2011) split the subfamily into Pitcairnioideae s.s. and five new subfamilies: Brocchinioideae, Lindmanioideae, Hechtioideae, Navioideae, and Puyoideae.

Pitcairnioideae s.s. consists of five genera: *Pitcairnia*, *Fosterella*, *Deuterocohnia*, *Dyckia*, and *Encholirium* (Givnish et al., 2011; Schütz et al., 2016). Based on sequences of eight plastid loci for placeholder species (Givnish et al., 2011), *Pitcairnia*, then *Fosterella* are sister to *Deuterocohnia* plus *Dyckia‐Encholirium*; several studies based on either plastid or nuclear sequences show that *Dyckia* is monophyletic only if *Encholirium* is included within it (see below). *Deuterocohnia*, *Dyckia*, and *Encholirium* form the so‐called Xeric Clade (Givnish et al., 2011), supported by numerous DNA substitutions and morphological traits (Givnish et al., 2011; Schütz et al., 2016; Gomes‐Da‐Silva et al., 2019). The smallest pitcairnioid genus—*Deuterocohnia*, with 17 accepted species (Schütz, 2013; Gouda and Butcher, 2024)—presents an outsized case for detailed study. All species are terrestrial, possess tough succulent leaves with CAM photosynthesis, and appear adapted to dry and/or high‐elevation habitats (Figure 1; Schütz, 2013; Crayn et al., 2015). Species of *Deuterocohnia* encompass an extraordinary range in elevation (0–3900 m a.s.l.), growth form (tiny cushion shrubs to massive rosette herbs with leaves up to 1 m long), and geographic distribution (single localities to 25° of latitudinal range). Most species of *Deuterocohnia* are restricted to the Andes of Bolivia and Argentina, with one species (*D. longipetala*) also found in Peru and another (*D. chrysantha*) being endemic to coastal Chile (Figure 2).

**Figure 1:**
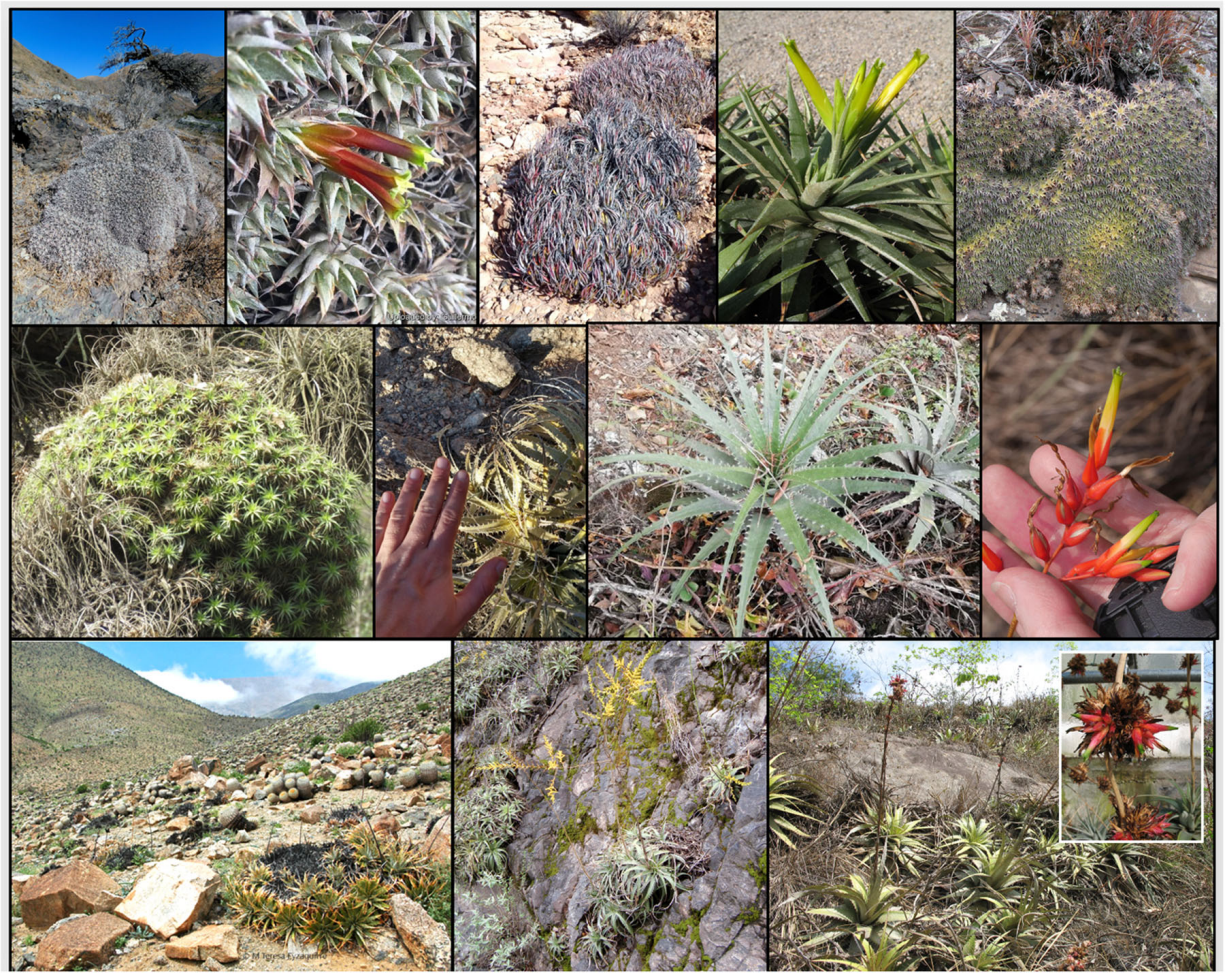
Representative species of *Deuterocohnia*. Top row—cushion shrubs from three lineages native to high elevations in the Andes: *D. abstrusa* (photograph by Francisco Cornell); reddish flowers of closely related *D. lotteae* (Guillermo Rivera); *D. strobilifera* (F. Cornell); *D. scapigera* (Timm Stolten); and *D. brevifolia* (Nicole Schütz). Middle row—taller, less compact growth forms from middle to low elevations: *D. sanctae‐crucis* (Richard C. Hoyer/Birdernaturalist); *D. schreiteri* (Leonel Roget); large rosettes of wide‐ranging *D. meziana* (Jose Balderrama); and flowers of *D. meziana* (R. Ripley). Bottom row—large rosettes at low elevations: *D. chrysantha* (María Teresa Eyzaguirre Philippi) from the Atacama Desert (note ring‐formation due to lateral branching); *D. longipetala* (Stefan Dressler); and *D. brevispicata* (N. Schütz), with inset showing perennial inflorescence on the type plant (T. Stolten).

**Figure 2:**
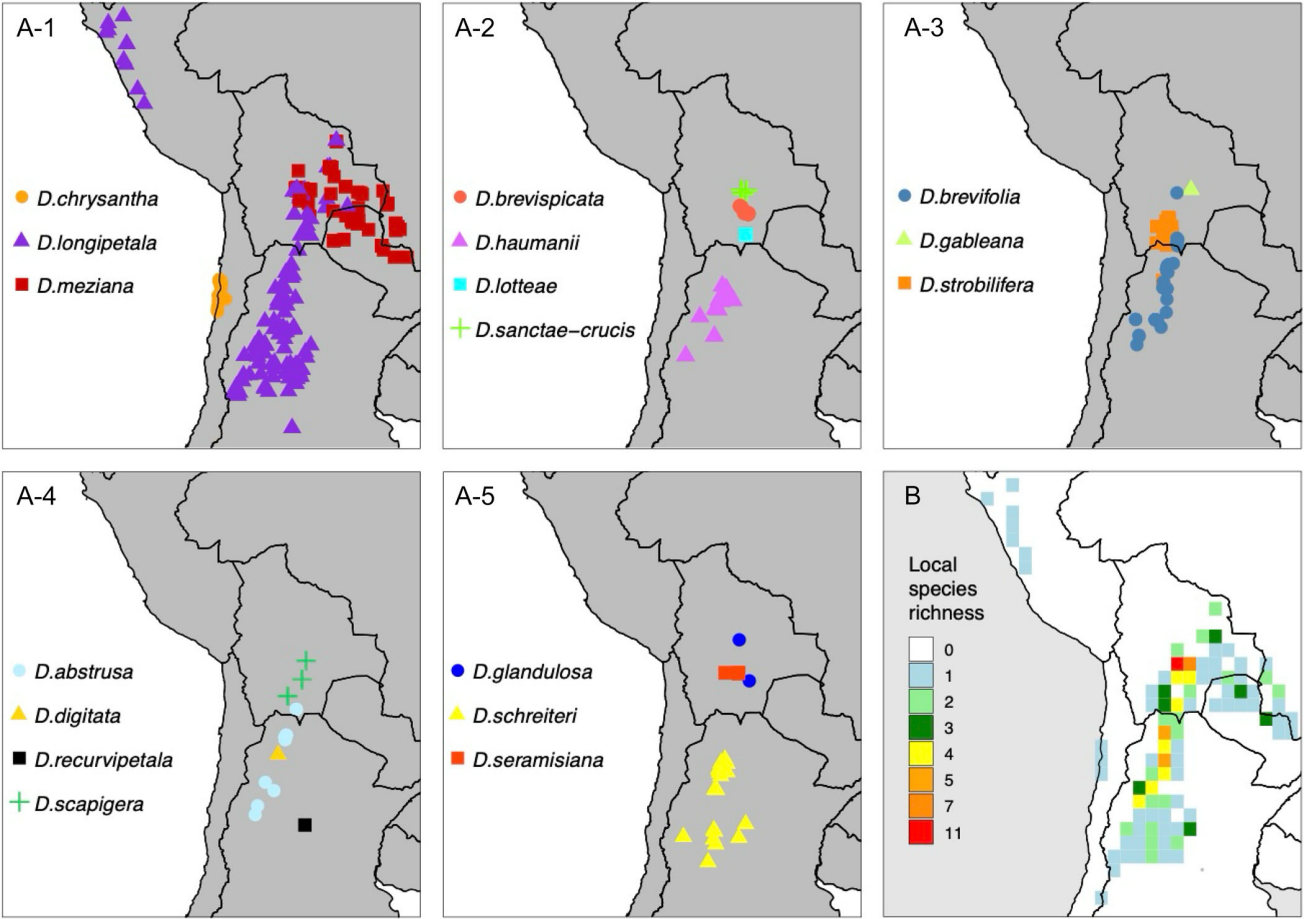
(A‐1–A‐5) Distribution of *Deuterocohnia* species. Data were extracted from GBIF. Species are grouped for clear presentation of multiple taxa on single panels, not to indicate phylogenetic relationships. (B) Species richness summarized in 1° × 1° grid.

Three species have especially interesting distributions. *Deuterocohnia longipetala*, by far the widest‐ranging species, has a disjunct distribution in the central Andes and northern Peru (Figure 2A) (Schütz, 2013). *Deuterocohnia chrysantha* is endemic to coastal areas of northern Chile (Zizka, 2003), and *D. meziana* ranges into the lowlands of western Brazil and northern Paraguay (Figure 2A). Some species (e.g., *D. gableana*, *D. recurvipetala*) are known only from their type localities, and many others have very narrow ranges in the central Andes (Schütz, 2013; GBIF.org, 2024). Several species co‐occur in a narrow region in southern Bolivia and northern Argentina (Figure 2A–E) with a center of diversity there (Figure 2F), so a key question is whether any of them originated via hybridization.

*Deuterocohnia* species are xerophytes with several adaptations to dry conditions, including tough, evergreen, succulent leaves with spiny leaf tips and margins, dense indumentum, massive adaxial hydrenchyma (water storage tissue), and CAM photosynthesis (Varadarajan and Gilmartin, 1988; Horres and Zizka, 1995; Givnish et al., 2007; Schütz, 2013; Crayn et al., 2015). All have lateral branching and with age form clonal, densely packed ring‐like colonies or (especially at high elevations) wide, low, compact cushions.

In coastal Chile, *D. chrysantha* grows in the Atacama, the driest nonpolar desert on Earth, adjacent to the cold Humboldt current upwelling offshore. Most *Deuterocohnia* species are native to arid and semiarid regions with dry seasons 4–9 months long; many grow on rocks, in open shrublands, and in montane dry‐forest understories in valleys and dry slopes of the eastern Andes (Schütz, 2013). In northwestern Argentina, *D. haumanii* and *D. schreiteri* dominate the local treeless plant communities, forming one of the very few bromeliad‐dominated vegetation types (Smith, 1964). Four species (*D. abstrusa*, *D. brevifolia*, *D. lotteae*, *D. scapigera*) were previously segregated in *Abromeitiella* and form extensive cushions with one to few flowers per rosette in the high Andean Puna (Schütz, 2013). These were sunk in *Deuterocohnia* based on continuity of morphology (Spencer and Smith, 1992), a decision confirmed by AFLP data (Horres, 2003) and sequences for five plastid and two nuclear loci (Schütz, 2013). All six species that range up to or above 2700 m (including the four former members of *Abromeitiella* and *D. digitata* and *D. strobilifera*), form cushions at those elevations, although those formed by *D. digitata* and *D. strobilifera* are aerodynamically somewhat rougher, with longer incurved leaves and a less smooth outline. Habitats occupied by the genus include fog deserts, matoral, lowland and montane savannas, chaco, thorn forest, montane dry forests, rock outcrops, and puna (Schütz, 2013).

*Deuterocohnia* offers a potential window into morphological evolution in response to elevation. Species from higher elevations are usually more compact, with shorter, fewer‐flowered, more erect inflorescences, shorter stems, shorter leaves, and more tightly packed clonal rosettes (Schütz, 2013), although formal statistical analysis and phylogenetic reconstructions have yet to been done. The more compact growth forms might represent adaptations to effectively drier conditions caused either by lower rainfall and warm temperature or by cold temperatures that reduce water and nutrient uptake by roots (Givnish, 2016). Leaves of several species in dry, rocky, or high‐elevation habitats are highly recurved or have red pigmentation, which might reduce water loss by increasing self‐shading, and at the same time protect against photoinhibition, UV damage, or frost. Flowers of *Deuterocohnia* species vary from inconspicuous greens and yellows, presumably associated with insect pollination, to red, orange, or bicolored flowers (Figure 1), often at lower elevations, pollinated by hummingbirds (Schütz, 2013; Schütz et al., 2016). Inflorescences range from sessile or short with single flowers, to massive, branched structures up to 2 m tall in *D. meziana*. The latter is woody and shrublike and possesses a cambium‐like meristematic cylinder (Benzing, 2000), which enables the plants to produce flowers repeatedly at the same inflorescence over many years. The formation of woody, perennial inflorescences is typical for most *Deuterocohnia* species and is an unusual character within Bromeliaceae, otherwise seen only in *Dyckia* and *Puya* (Benzing, 2000).

Schütz (2013) and Schütz et al. (2016) reconstructed the first detailed plastid and nuclear phylogenies for *Deuterocohnia* using DNA sequences of three chloroplast intergenic spacers (*rpl32*‐*trnL*, *rps16*‐*trnK*, *trnS*‐*ycf3*) and two nuclear single‐copy genes (PHYC exon 1, PRK exons 2–5). Several species from each of the five genera of Pitcairnioideae were sampled, including all 17 *Deuterocohnia* species, with almost all of those represented by multiple samples per species in the plastid analysis. *Pitcairnia* and *Fosterella* were used as outgroups by Schütz (2013), given their placement sister to the rest of Pitcairnioideae by Givnish et al. (2011) based on sequences of eight plastid loci. Additional outgroups from Bromelioideae, Hechtioideae, Puyoideae, and Tillandsioideae were employed by Schütz et al. (2016). Maximum likelihood analyses of relationships in *Deuterocohnia* produced plastid and nuclear trees with many unresolved nodes (e.g., 58 of 102 in the plastid analysis of Schütz, 2013) and widespread low support. Most species appeared to be polyphyletic in the heavily sampled plastid ML tree; Bayesian analysis of the same data, however, resolved many *Deuterocohnia* species as monophyletic (Schütz, 2013). There was a pronounced cytonuclear conflict, with *Deuterocohnia* Clade B in Schütz's plastid tree (*D. brevispicata*, *D. gableana*, *D. meziana*, *D. scapigera*, *D. seramisiana*) being sister to a clade of *Dyckia* intermingled with *Encholirium*, with these clades sister to plastid *Deuterocohnia* Clade A. *Deuterocohnia* was, however, clearly monophyletic in both the PHYC tree and combined nuclear trees (Schütz, 2013; Schütz et al., 2016). While only a few species were resolved as monophyletic in the plastid tree, Schütz (2013) discovered a striking correspondence between plastid clades and the geographic areas from which different samples were obtained, suggesting hybridization/introgression and resulting chloroplast capture. Thus, the five species belonging to Schütz's Clade B were all found north of the Rio Pilcomayo in Bolivia, whereas the remaining 12 species, belonging to Clade A, were south of it. Schütz (2013) therefore proposed that the Rio Pilcomayo might have been an obstacle to dispersal when swollen with glacial meltwater during interglacial periods. However, her data suggested chloroplast capture from *Dyckia*‐*Encholirium* roughly 11 million years ago, well before the Pleistocene.

Gomes‐Da‐Silva et al., (2019) used parsimony to produce a phylogeny for the Xeric Clade based on morphology and sequences of five plastid genes/spacers and two single‐copy nuclear genes. That phylogeny indicated that *Deuterocohnia*, *Dyckia*, and *Encholirium* were all para‐ or polyphyletic. *Dyckia* consistently emerges as monophyletic if *Enchlorium* is included within it (Krapp et al., 2014; Schütz et al., 2016; Gomes‐Da‐Silva et al., 2019). However, Gomes‐Da‐Silva et al., (2019) concatenated plastid and nuclear DNA sequences despite their discordance regarding the para‐ vs. monophyly of *Deuterocohnia* (Schütz et al., 2016), used parsimony analyses that are less powerful and less justified than maximum likelihood, and presented no analyses based on nuclear sequences alone, so their conclusions regarding relationships of *Deuterocohnia* species to each other are questionable.

The widespread lack of resolution and support in these plastid and nuclear trees reflects the notoriously low rates of molecular evolution across Bromeliaceae (Givnish et al., 2007, 2011, 2014) and precludes any detailed analysis of cytonuclear conflict at the species level. Phylogenetic resolution and support for recent or slowly evolving groups can be improved by using hypervariable DNA markers. Consistent with that possibility, Zenk et al. (2018) were able to clearly separate two subspecies of *D. meziana*, as well as *D. brevispicata*, *D. seramisiana*, and *D. longipetala*, all with limited admixture, in a structure analysis using 15 hypervariable microsatellite loci.

Today, phylogenomic approaches have become the gold standard for phylogenetic analyses of recently evolved groups by sequencing the whole plastid genome and numerous single‐ or low‐copy nuclear genes (Moore et al., 2007; Barrett et al., 2014; Givnish et al., 2010, 2018; Johnson et al., 2019; Kriebel et al., 2019; Leebens‐Mack et al., 2005, 2019; Baker et al., 2022; Timilsena et al., 2022; Karimi et al., 2024; Rose et al., 2025). Introns and intergenic spacers in both genomes evolve faster than exons and can greatly aid phylogenetic reconstruction but can be hard to align across more distant relatives. Target sequence capture (also known as target enrichment) use RNA or DNA probes to enrich sequencing libraries for specifically targeted loci (Faircloth et al., 2012; Lemmon et al., 2012; Weitemier et al., 2014; Baker et al., 2022). This approach, combined with next‐generation sequencing, can cost‐effectively produce DNA sequences for hundreds to thousands of targeted nuclear loci, and often retrieves plastid genome sequences due to the abundance of unenriched plastid DNA. Although recent sequencing probes are more effective in targeting the designated regions, using off‐target reads to build plastome phylogenies are still effective in recent phylogenomic studies (i.e., Schneider et al., 2021; Thureborn et al., 2024; Rose et al., 2021a, 2025). Target sequence capture of nuclear loci also provides powerful and independent lines of evidence regarding ancestry. Nuclear loci generally evolve faster than plastid loci (Wolfe et al., 1987; Drouin et al., 2008), and their scattering across the vast nuclear genome provides numerous unlinked markers of evolutionary history—unlike plastid genes, which are inherited as a single unit, making plastid phylogenies subject to distortion via chloroplast capture following hybridization (e.g., Baldwin et al., 2023).

The recent avalanche of phylogenetically informative data produced by target sequence capture has dramatically increased our ability to resolve relationships among closely related or slowly evolving species. Target sequence capture using the Angiosperm353 bait kit (Johnson et al., 2019; Baker et al., 2022; Perez‐Escobar et al., 2024) has been particularly transformative in allowing rapid reconstruction of relationships at all levels. However, the Angiosperm353 baits are based on slow‐evolving exons that are highly conserved across angiosperms and are not especially useful in resolving relationships among closely related or very slowly evolving groups. It is thus not surprising that studies of bromeliad relationships using Angiosperm353 often provide weak support for relationships, as seen in recent studies of Bromelioideae (Bratzel et al., 2023) and Puyoideae (Aguirre‐Santoro et al., 2024).

To deal with slow evolutionary rates in Bromeliaceae, Yardeni et al. (2022) developed the Bromeliad1776 bait kit that targets 1776 single‐copy nuclear genes (SCGs), which produced a strongly supported nuclear phylogeny focused on subfamily Tillandsioideae. Yardeni et al. found greater support values for individual gene trees based on their custom loci vs. the Angiosperm353 loci, and there were nearly six times as many assembled loci. However, their kit is based on genome sequences from only two subfamilies (Tillandsioideae and Bromelioideae), so there is potential for taxon bias in sequence recovery in other subfamilies.

Here, we produced shallow genome sequences for representatives of all other bromeliad subfamilies to create a new Bromeliad1815 kit, designed to conduct target sequence capture across all Bromeliaceae without bias. We then applied this kit to reconstruct nuclear and plastome phylogenies for *Deuterocohnia*, calibrate them against time, assess the evidence for hybridization or incomplete lineage sorting, and evaluate patterns of morphological evolution in relation to elevation and history of geographic spread in South America. We also tested whether target sequence capture can be used effectively on tissue samples from herbarium specimens in bromeliads, as it has been in other angiosperm families (Baker et al., 2022), and whether our nuclear data are consistent with relationships among bromeliad subfamilies based on plastid sequences (Givnish et al., 2011, 2014) or with those obtained using the Bromeliad1776 or Angiosperms353 nuclear loci (Yardeni et al., 2022).

## MATERIALS AND METHODS

### Bait design and sequencing

We modified a bait kit adapted from the Bromeliad1776 kit (Yardeni et al., 2022), which itself was based on whole‐genome assemblies of *Ananas* (Ming et al., 2015) and *Tillandsia* (de La Harpe et al., 2020). To capture genomic diversity across all bromeliad subfamilies, seven additional bromeliad genomes were sequenced by Novogene using paired‐end 150‐bp Illumina NovaSeq reads. We assembled genomes using MarSuCa version 4.0.9 (Zimin et al., 2013) for *Brocchinia acuminata*, *B. paniculata*, and *B. reducta* (Brocchinioideae), *Lindmania longipes* (Lindmanioideae), *Hechtia lundelliorum* (Hechtioideae), *Navia splendens* (Navioideae), and *Pitcairnia atrorubens* (Pitcairnioideae). Assembled genome sizes ranged from 278.0 to 364.8 Mb, with estimated coverages of 64.1–103.9*x* (Table 1). We ran BUSCO version 1.0.0 (Di Tomasso et al., 2017) to assess the quality of these assembled genomes using the Poales BUSCO library with 4896 benchmarking universal single copy orthologs (BUSCO genes). We retrieved >75% of the BUSCO genes (with ca. 5% fragmented) for all assembled genomes (Table 1), indicating a relatively high quality of the genomes assembled. Links to draft genomes uploaded to CoGe are given in Appendix S1. We also included published genome sequences for *Puya raimondii* (Liu et al., 2021) of Puyoideae and *Ananas comosus* (Ming et al., 2015) of Bromelioideae. Although we did not include any tillandsioid sequences, the original Bromeliad1776 bait kit included extensive sampling from *Tillandsia* (Yardeni et al., 2022).

**Table 1:** Assembly metrics for newly sequenced bromeliad genomes. N50 is the contig length such that 50% of the total assembly length is contained in contigs at least that long. Also shown are the count of the assembled contigs, the total number of assembled bases, coverage depth (number of bases sequenced in the raw Illumina reads divided by the total bases assembled), estimated genome size (C), and the proportion of guanine and cytosine bases in the assembly.

							% BUSCOs:		
Species	N50	Count	Assembled bases	Coverage (x)	Estimated C	%GC	Complete	Fragmented	Missing
*Brocchinia acuminata*	7330	91,420	283,960,250	96.2	338,520,09	38.8	78.9	4.6	16.5
*Brocchinia paniculata*	14,730	73,763	278,070,607	83.5	442,065,033	38.1	81.8	4.6	13.6
*Brocchinia reducta*	5475	130,651	298,905,014	76.6	397,842,700	39.6	75.5	5.6	18.9
*Hechtia lundelliorum*	10,079	92,276	267,180,485	103.9	345,720,649	38.9	81.1	4.9	14.0
*Lindmania longipes*	7408	125,226	279,097,171	74.8	410,422,751	39.3	79.2	4.7	16.1
*Navia splendens*	3637	338,502	364,778,676	64.1	846,954,173	43.4	75.7	5.6	18.7
*Pitcairnia atrorubens*	6407	137,752	350,553,384	74,9	409,181,016	38.9	78.7	=4.6	16.7

Using HybPiper version 1.3 (Johnson et al., 2016), we assembled the target sequences for the 1776 genes identified by Yardeni et al. (2022) from raw reads cleaned by Fastp version 0.12.4 (Chen et al., 2018). Sequences of each gene were aligned using MAFFT version 7.490 (Katoh et al., 2002) and trimmed using TrimAl version 1.4.1 (Capella‐Gutiérrez et al., 2009). We then inferred individual gene trees for each gene across the nine WGS species (see above) as well as the original *Ananas* reference using maximum likelihood in IQ‐Tree version 2.0.7 (Minh et al., 2020a), collapsing branches with ultrafast bootstrap support (UF BS) below 70% using GoTree version 0.4.4 (Lemoine and Gascuel, 2021). We discarded 170 loci that produced unresolved trees as uninformative; 1305 loci were considered informative because they yielded trees with more than four nodes with ≥90% UF BS. We calculated pairwise difference scores using Mothur version 1.46.1 (Schloss et al., 2009) for assembled target sequences at the locus level. We then selected 351 target sequences that exhibited ≥15% divergence from the corresponding sequences for *Ananas comosus*, which was used as the basis for the Bromeliad1776 kit design. According to Arbor Biosciences (2020), baits can capture target sequences with up to 10–15% divergence if capture is conducted at 62°C. We then identified a total of 209 overlapping sequences between the 1305 informative loci and the 351 sequences even more divergent from *Ananas*. Those 209 overlapping sequences were added into our modified kit; 168 of these came from *Brocchinia* (see Appendix S2 for list of deletions and additions made in moving from the 1776 kit to the 1815 kit). Our new Bromeliad1815 bait kit comprises 1815 loci (57,000 baits) and is tailored to capture sequences more effectively beyond Bromelioideae and Tillandsioideae. The kit was tested and manufactured by Daicel Arbor Biosciences (Ann Arbor, MI, USA).

### Plant materials, DNA extractions, and library preparation

This study is focused primarily on clarifying relationships among species within *Deuterocohnia* and secondarily on relationships among genera of Pitcairnioideae and subfamilies of Bromeliaceae. The ingroup included 19 samples and all 17 species of *Deuterocohnia*, provided by Nicole Schütz, Kurt Weising, Georg Zizka, and the Botanical Garden of Heidelberg (Heidelberg, Germany) as DNA extracts from silica‐dried or fresh tissues (Table 2). Single samples were chosen arbitrarily among those collected by Schütz (2013) to represent each species, with a second sample of *D. longipetala* added to represent the different positions of Argentine samples in Schütz's chloroplast tree. We also sequenced 26 samples of *Pitcairnia*, nine of *Brocchinia*, and three of *Navia* (Appendix S3). Of these, 10 *Pitcairnia*, four *Brocchinia*, and two *Navia* were extracted from herbarium snippets provided by the New York Botanical Garden Herbarium (NYBG) and the Wisconsin State Herbarium (WIS) (Appendix S3). DNA was extracted from herbarium tissue samples using two 6% CTAB reactions (Smith et al., 1991) per sample, with the resulting extracts being subsequently pooled to increase yield. CTAB DNA extractions were also obtained from silica‐dried tissues that were freshly gathered in the University of Wisconsin‐Madison greenhouse for one *Pitcairnia* and two *Brocchinia* samples.

**Table 2:** Accession list of ingroup *Deuterocohnia* Species, with two accessions of *D. longipetala* labeled as 1 and 2. Except for *D. strobilifera* (from the Botanical Garden Heidelberg, Germany), all samples were sourced from Schütz (2013). Appendix S1 provides detailed assembly quality for both ingroups and the outgroup. “No. raw Reads” indicates the number of paired reads in uncleaned data. “No. nuclear genes” reflects the number of nuclear genes in the MAFFT‐generated trimmed alignment. “% Missing in nuclear alignment” shows the percentage of missing bases; samples with >50% missing data were excluded from phylogenetic analysis (Y = included, N = excluded). “GetOrganelle assembly coverage” denotes coverage per base, indicating if a full plastome was generated (Y = yes, N = no). “% Missing in plastome alignment” shows the percentage of missing bases in the trimmed plastome alignment, with >50% missing leading to exclusion (Y = included, N = excluded). “No. plastid exons” lists the number of plastid exons in the trimmed alignment, with the corresponding missing percentage; samples with >50% missing were excluded (Y = included, N = excluded).

Species	No. raw reads	No. nuclear genes	% Missing in nuclear alignment (Inclusion in nuclear tree)	GetOrganelle assembly coverage (If full plastome assembled)	% Missing in plastome alignment (Inclusion in full plastome tree)	No. plastid exons	% Missing in plastid exon alignment (inclusion in plastid exon tree)
*D. sanctae‐crucis*	8,009,364	1747	0.08 (Y)	260.7 (Y)	0 (Y)	74	0 (Y)
*D. longipetala 1*	14,881,280	1752	0.08 (Y)	273.6 (N)	0.98 (N)	23	0.58 (N)
*D. lotteae*	10,574,672	1758	0.08 (Y)	250.8 (Y)	0.01 (Y)	74	0 (Y)
*D. brevifolia*	18,276,061	1760	0.08 (Y)	21.1 (N)	0.28 (Y)	60	0.05 (Y)
*D. scapigera*	26,772,737	1765	0.07 (Y)	201.9 (N)	0.97 (N)	9	0.95 (N)
*D. recurvipetala*	21,788,127	1773	0.07 (Y)	18.8 (N)	0.12 (Y)	67	0.02 (Y)
*D. glandulosa*	11,083,944	1755	0.09 (Y)	92.7 (Y)	0.03 (Y)	74	0 (Y)
*D. brevispicata*	9,814,181	1752	0.08 (Y)	104.6 (N)	0.07 (Y)	74	0 (Y)
*D. seramisiana*	7,076,017	1740	0.09 (Y)	30.8 (N)	0.1 (Y)	68	0.03 (Y)
*D. abstrusa*	5,788,288	1731	0.09 (Y)	104.9 (Y)	0.01 (Y)	74	0 (Y)
*D. strobilifera* var. *inermis*	6,177,251	0	1 (N)	Failed	1 (N)	0	1 (N)
*D. haumanii*	9,323,344	1752	0.08 (Y)	63.5 (N)	0.08 (Y)	74	0 (Y)
*D. digitata*	7,495,341	1744	0.08 (Y)	94 (N)	0 (Y)	74	0 (Y)
*D. schreiteri*	7,910,420	1741	0.09 (Y)	118.3 (N)	0 (Y)	74	0 (Y)
*D. longipetala 2*	20,383,478	1763	0.08 (Y)	339.3 (N)	0.99 (N)	17	0.82 (N)
*D. gableana*	8,082,330	1737	0.08 (Y)	31.5 (N)	0.2(Y)	70	0.01 (Y)
*D. meziana*	10,877,576	1756	0.09 (Y)	58.1 (N)	0.02 (Y)	74	0 (Y)
*D. brevifolia*	4,054	7	1 (N)	Failed	1 (N)	0	1 (N)
*D. chrysantha*	8,076,217	1744	0.08 (Y)	339.9 (Y)	0 (Y)	74	0 (Y)
*D. strobilifera*	5,924,318	1730	0.09 (Y)	15.6 (N)	0.24(Y)	8	0.73 (N)

DNA samples were checked for quality and quantified using a Qubit fluorometer Broad Range Kit (ThermoFisher, Waltham, MA, USA). Library preparation, target capture, and sequencing were conducted by Daicel Arbor Biosciences. All but one sample were sequenced on an Illumina platform, with *Deuterocohnia strobilifera* sequenced on the Element Biosciences AVITI. All samples were analyzed, based on evidence showing high similarity and combinability across platforms (Landis et al., 2025). Samples extracted from herbarium snippets contained degraded, highly fragmented DNAs and underwent target capture and sequencing using the myReads degraded DNA NGS service package from Diacel Arbor Biosciences; the other samples were processed using the standard package.

To expand our data set using existing sequences, we downloaded reference plastomes, transcriptomes, and whole‐genome sequences from the Sequence Read Archive (SRA: Leinonen et al., 2011) for *Dyckia*, *Fosterella*, and additional *Deuterocohnia* samples. Nuclear and plastome data were assembled as described below; detailed statistics for individual samples are given in Appendix S3. Two species—*Deuterocohnia glandulosa* (SRR13700303) and *Fosterella penduliflora* (SRR13700302)—passed plastome assembly and were included in the plastome data set but failed nuclear assembly. Four *Dyckia* RNA‐sequencing data sets—*D. velascana* (SRR29188277), *D. remotiflora* var. *montevidensis* (SRR29188276), *D. niederleinii* (SRR29188275), and *D. leptostachya* (SRR29188274)—were included in the nuclear data set but also failed plastome assembly (Appendix S3). *Fosterella spectabilis* (SRR29188278) passed nuclear assembly but failed plastome assembly. We also used the bromeliad genomes we sequenced or downloaded to generate nuclear and plastid sequence data via in silico capture and plastome assembly (Appendix S3). Two species of *Typha* (*T*. *latifolia*, *T*. *domingensis*) were used as outgroups, based on Typhaceae, then Bromeliaceae being sister to all other members of the monocot order Poales based on nuclear DNA sequence data (Baker et al., 2022; Timilsena et al., 2022).

### Genome assembly

All Illumina sequences were trimmed to remove adaptor sequences, and quality control enforced with Fastp version 0.12.4 (Chen et al., 2018) and confirmed with FastQC v.0.11.7 (Andrews, 2010). We used HybPiper (Johnson et al., 2016) to assemble only the exon regions based on the 1815 targeted single‐copy reference genes and produce the nuclear data set.

We used reference‐guided de novo assembly to produce plastome sequences. Contigs were assembled using GetOrganelle version 1.7.7.1 (Jin et al., 2020), with parameters determined by multiple testing (Appendix S4). The primary contigs were then scaffolded in Geneious Prime 2023.2.1 (Dotmatics, Boston, MA, USA) by mapping them onto the reference plastome of *Puya mirabilis* (GenBank NC045380.1) without the second inverted repeat. We extracted the majority rule consensus sequences to build a data set of whole plastome assemblies. Contig ends were manually trimmed whenever misassemblies were identified, as indicated by high mismatch scores against the reference. The resulting complete or nearly complete plastome sequences were compiled in the plastome data set. We also extracted 74 plastid exons to produce the plastid exon data set.

### Alignment

Sequences from all three data sets were aligned separately using MAFFT v.7.490 (Katoh et al., 2002), and the alignments were trimmed with TrimAL version 1.4.1 (Capella‐Gutiérrez et al., 2009). For the nuclear and plastid exon data sets, we concatenated individual genes using SequenceMatrix version 1.9 (Vaidya et al., 2011). We checked the percentage of missing bases in the final alignments for all three data sets. For downstream analysis, we removed any samples with >50% missing data, except for the *Typha* nuclear sequences which have roughly 70% missing bases (Appendix S3).

### Phylogenomic analysis

For the nuclear data set, phylogenetic relationships were inferred by IQ‐Tree2 version 2.2.2 (Minh et al., 2020a) via maximum likelihood (ML) applied to the concatenated data, using a partitioned analysis with a greedy strategy (‐m MFP + MERGE) to account for different substitution rates across genes (Lanfear et al., 2012; Chernomor et al., 2016). Ultrafast bootstrapping scores (using the ‐bnni option to reduce the chance of overestimating support) and gene concordance factors (gCFs) were calculated on the derived nuclear ML tree (Minh et al., 2020b). To reconstruct a species tree consistent with the multispecies coalescence model, we inferred individual ML gene trees using IQ‐Tree2 after removing one gene represented in ≤3 samples. We inferred the species tree based on the remaining 1814 genes using ASTER v1.22 (weighted‐ASTRAL hybrid mode), which assesses the impact of quartets considering both bootstrap supports and branch length (Zhang et al., 2018, 2022).

### Time calibration of phylogenies

Because our nuclear data set contains more than 2.5 million bases, we first selected 100 clock‐like nuclear genes with SortaDate (Smith et al., 2018) to make the calibration feasible. We constrained the topology of the nuclear chronogram using the ML nuclear tree and ran 112 million MCMC generations in BEAST2 (Suchard et al., 2018). For the plastid exon and full plastome data sets, we used BEAST2 v.2.7.7 in CIPRES (Miller et al., 2015) to estimate Bayesian trees on the full data sets, running each with 80 million MCMC generations. For all three data sets, we used optimized relaxed molecular clock, birth and death model, and GTR + I + R4 substitution model with estimated substitution rates with parameters determined using by IQ‐tree2 ModelFinder (Kalyaanamoorthy et al., 2017). Two secondary calibration points were used to calibrate the phylogenies using normally distributed priors, setting the crown age of Bromeliaceae as 19.1 ± 3.4 SD Mya and the stem age as 100.0 ± 5.2 SD Mya (Givnish et al., 2011). All BEAST phylogenies were summarized after 25% burn‐in percentage; convergence of run parameters was achieved in each calibration run.

### Reticulate evolution

We used tree simulation approaches to evaluate the relative importance of incomplete lineage sorting vs. gene flow (i.e., hybridization and/or introgression) in creating cytonuclear discordance at individual branches. We conducted 5000 replicates using the sim.coal.mpest function in the R package Phybase v.2.0 (Li and Yu, 2010) to simulate gene trees under the multispecies coalescence (MSC) model with the ASTRAL tree as the true species tree. Nonparametric bootstrap support was then calculated for both the nuclear maximum likelihood (ML) tree and the BEAST‐derived plastome tree using the SumTrees function in the Python package DendroPy v.5.0.1 (Moreno et al., 2010; Sukumaran and Holder, 2010). This analysis summarized the proportion of simulated gene trees under the MSC model that support a given clade in the full plastome tree. High support values (>0.9) suggest that incomplete lineage sorting (ILS) is likely the dominant process causing cytonuclear conflict. Conversely, lower support values may indicate that other evolutionary processes, such as hybridization or plastome capture, play a more significant role in explaining the cytonuclear conflicts, while ILS is still likely to happen. The same or similar approach has been applied to assess whether hybridization/introgression and plastome capture, besides ILS, produces patterns of reticulate evolution (Folk et al., 2017; Cai et al., 2021; Baldwin et al., 2023; Rose et al., 2021b, 2025).

For further assessments of hybridization within *Deuterocohnia*, we used SnaQ (Solís‐Lemus and Ané, 2016) and corresponding functions in PhyloNetwork (Solís‐Lemus et al., 2017) to reconstruct phylogenetic networks. To determine the optimal number of hybridization events, we first reconstructed networks with a maximum of 0, 1, 2, 3, or 4 reticulations (hmax = 0–4), using 20 runs for each case and then compared negative log‐likelihoods. Negative log‐likelihoods had a steep drop from hmax = 1 to 2 and then stayed nearly constant for hmax = 3 and 4, suggesting that the best model was hmax = 2. We accordingly constrained the maximum number of reticulations to hmax = 1 or 2, and conducted 10 runs and 30 replicates to estimate networks. We compared the log likelihood score and networks to identify the biologically meaningful networks (Solís‐Lemus and Ané, 2016).

### Trait and elevational data, geographic distribution, phylogenetic regression, and PCA

Data for morphological traits, growth habit, and elevation for *Deuterocohnia* species were obtained from Schütz (2013). We downloaded and cleaned distribution records for *Deuterocohnia* from GBIF.org. Schütz (2013) designated *D. sanctae‐crucis* as distinct from *D. scapigera* and one misclassified *D. digitata* occurrence in Bolivia (corrected to *D. sanctae‐crucis*). We followed the revision of vouchers by Schütz (2013); three misidentified *D. chrysantha* from the Andes were removed. Phylogenetically structured PCA (pPCA; Polly et al., 2013) using the R package PhyTools v.4.3.3 (Revell et al., 2012) and non‐phylogenetically structured PCA were conducted using both quantitative and categorical traits. Categorical traits were encoded as ordinal values (“0”, “1”, “2”), where each number represents a distinct category without implying any numerical order between them. We based the pPCA on the dated nuclear phylogeny derived from BEAST for *Deuterocohnia* species only. We used the dated nuclear phylogeny to conduct phylogenetically structured regression (pGLM) to understand how each quantitative trait responds with changes in elevation. We included PC1 and PC2 from both pPCA and PCA into pGLM.

We applied backward elimination starting from the full model with elevation, precipitation, and temperature with all possible interaction terms to identify the best model with only fixed effect. The best model for each trait variable was selected with the lowest AIC score. If the best models based on AICs contained multiple interaction terms, 3‐fold and 4‐fold cross‐validation were used to assess overfitting, and the final model was chosen based on AIC, mean squared error from cross validation, and parsimony. The best model with fixed effects was tested with two random effects from species alone (NP) and species with phylogenetic structure (P) using the R package phyr v.1.1.0 (Ives et al., 2020), with a cutoff value of 0.5 (Ives and Helmus, 2011). If *P* < 0.5, we chose the model with phylogenetic influence. A cut‐off of 0.5 was chosen given we aimed to include any phylogenetic structure if possible, and the same method was implemented by Smith et al. (2023). For the selected model, residual normality was checked, and total and partial R^2^ values were calculated using the R2_lik function in the R package rr2 v.1.1.1 (Ives and Li, 2018), and the Yekutieli–Benjamini–Hochberg procedure (Yekutieli and Benjamini, 1999) was applied to adjust for false discovery rate in multiple tests (R Core Team, 2023). We overlaid mean elevation, PC1 and PC2 scores from regular PCA, and mean latitude and longitude on the dated nuclear phylogeny using the contMap function of PhyTools v.4.3.3 (Revell et al., 2012), which reconstructed ancestral state based on maximum likelihood.

### Biogeographic and ecological reconstruction

To reconstruct historical biogeography, we calculated the geographic centroid (mean latitude and longitude) of GBIF records for each species, then inferred ancestral centroids using maximum likelihood. The narrow geographic range of *Deuterocohnia* within the western hemisphere and our use of negative latitudes within the southern hemisphere (grading continuously into positive values in moving across the Equator) allowed us to avoid issues of discontinuity and periodicity. We chose this approach—rather than more powerful analyses using BioGeoBears (Matzke, 2013, 2025)—given the small area to which all species are restricted, and the lack of previously determined areas of endemism at fine scales into which species distributions could be atomized informatively. Aagesen et al. (2012, 2013) identify such areas based on plant distributions in the southern Andes, but they cannot be used because several overlap with each other, violating the assumptions of BioGeoBears. Reconstructions of character‐state and biogeographic evolution have frequently used centroids (see Paradis et al., 2004; Lemey et al., 2010; Quintero et al., 2015; Marcussen and Meseguer, 2017), but the results can be misleading for species with disjunct or non‐convex ranges (applies only to *Deuterocohnia longipetala* here), and ML analyses overlook details of dispersal, extinction, and vicariance that BioGeoBears was designed to assess.

We were able to use BioGeoBears v.1.1.3 (Matze, 2013, 2025) to estimate ancestral habitats for *Deuterocohnia*. Following Schütz (2013), we scored the presence of each species in 14 different habitat types (“ecoregions”) drawn from the world classification of Dinerstein et al. (1995). To make calculations tractable, we excluded the seven habitats occupied by only one species in addition to at least one other habitat; such autapomorphic habitats are unlikely to have been ancestral. We compared model fits for DEC, DEC + J, DIVAlike, DIVAlike + J, BAYAREAlike, and BAYAREAlike + J based on log likelihood ratios and AIC values. Based on the results, DEC was the preferred model (Appendix S5). We used stochastic mapping (50 replicates) to tally the average number of shifts between each pair of habitats. We also indirectly assessed habitat shifts by using maximum likelihood to overlay elevation—a proxy for several Andean and circum‐Andean habitats—on the nuclear phylogeny (see above).

## RESULTS

### Sequencing and assembly

For samples extracted from fresh or silica‐dried leaf tissue, we recovered an average of 1534 and 1363 nuclear genes at 50% and 75% of the reference length, respectively (Appendix S3). These averages dropped to 547 and 434 genes for herbarium samples, with useful data retrieved for only 5 of 16 samples (31%). After poorly aligned regions were trimmed, fresh or silica‐dried samples averaged 14% missing data in the nuclear alignment and 23% in the plastome alignment. All herbarium samples averaged 67% missing data in the nuclear alignment and 81% in the plastome alignment (Appendix S3). Among the five herbarium samples from which nuclear sequencing data were successfully retrieved, on average 1577 and 1345 nuclear genes at 50% and 75% of the reference length were recovered with on average 11% missing nuclear data and 41% missing full plastome data.

After we excluded samples with >50% missing data, the final nuclear alignment contained 55 species and 59 samples, including all 17 *Deuterocohnia* species and 38 outgroup species (Appendix S3), for a total of 2,738,053 aligned bases with 10% missing data. The final plastome alignment—after excluding one copy of the inverted repeat, misassembled or misaligned regions, and samples with >50% missing data—totaled 109,038 aligned bases with 7.7% missing data. The plastome alignment included 15 species (16 samples) of *Deuterocohnia* and 37 species (41 samples) of outgroup taxa, with the latter including seven plastome sequences downloaded from GenBank and SRA (Appendix S3).

### Phylogenetic reconstruction and reticulation

**Figure 3:**
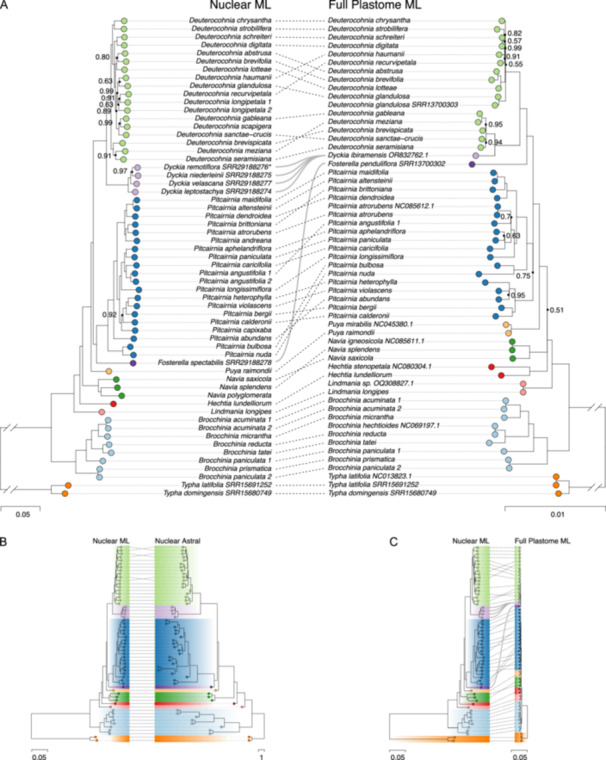
(A) Concatenated nuclear maximum likelihood (ML) tree vs. plastome ML tree; note differences in scale of branch lengths. Nodes with ultrafast bootstrap support values below 100 are labeled. Colors in all panels correspond to different genera. (B) Concatenated nuclear ML tree vs the ASTRAL species tree. (C) Concatenated nuclear ML tree and plastome ML tree, with branch lengths adjusted to represent substitution rates on the same scale in both.

The nuclear maximum‐likelihood phylogeny based on the concatenated data is fully resolved and strongly supported, with only 10 of 58 nodes having bootstrap values (UF BS) < 100%, and only two of those having UF BS < 80% (Figure 3A). All bromeliad genera with multiple species sampled are retrieved as monophyletic with 100% UF BS; relationships among genera all have 100% UF BS except for that between *Fosterella* and *Pitcairnia* (92% UF BS). *Deuterocohnia* is resolved as sister to *Dyckia*, with both sister to *Pitcairnia* + *Fosterella* (Figure 3A). Based on our gCF scores (percentage of gene trees supporting a clade), nuclear backbone relationships are often moderately to weakly supported (33.8–76.7%), but with 100% support for the positions of *Dyckia* sister to *Deuterocohnia* and 96.8% support for *Brocchinia* sister to all other Bromeliaceae (Appendix S6). Several nodes within *Deuterocohnia*, *Dyckia*, and *Pitcairnia* have much lower gCFs (Appendix S6). The nuclear ASTRAL tree is congruent to the nuclear ML tree, with high quadripartition support values throughout (Figure 3B). Nuclear relationships among the six bromeliad subfamilies sampled are (Brocchinioideae, (Lindmanioideae, (Navioideae, (Hechtioideae, (Pitcairnioideae, Puyoideae))))) are identical to those found previously based on plastid sequences (Givnish et al., 2011) but not Bromeliad1776 or Angiosperms353 nuclear sequences (Yardeni et al., 2022).

**Figure 4:**
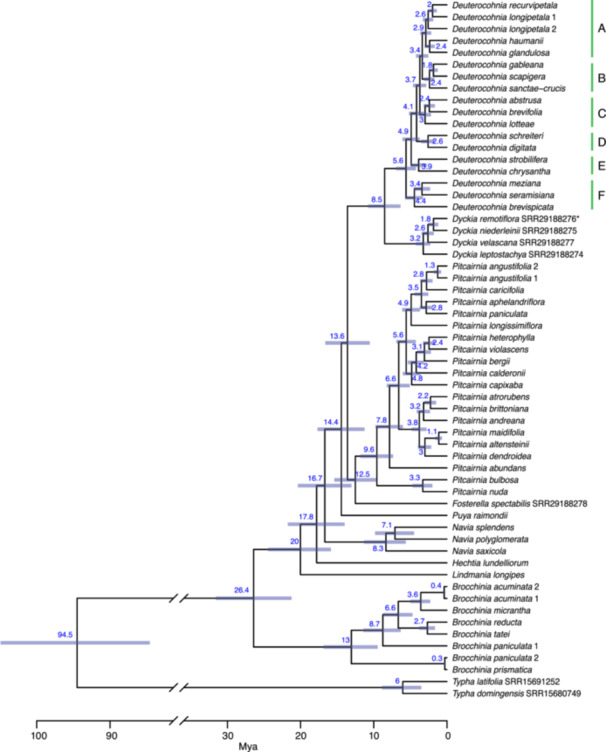
Dated ML nuclear phylogeny with estimated ages and error bars representing the highest posterior density (HPD) interval at the 95% level. All posterior probabilities of nodes are 1.0. Letters and bars indicate clades within *Deuterocohnia* recognized in text.

Within *Deuterocohnia*, six clades with 100% UF BS can be distinguished: (A) *recurvipetala*‐*longipetala*‐*haumanii*‐*glandulosa*, (B) *sanctae‐crucis* + *gableana‐scapigera*, (C) *lotteae* + *abstrusa*‐*brevifolia*, (D) *schreiteri‐digitata*, (E) *chrysantha‐strobilifera*, and (F) *brevispicata* + *meziana‐seramisiana* (Figures 3 and 4). Clades F, then E and D are sister to all other species of the genus. Clade C consists of three of the four species formerly placed in *Abromeitiella*, with the fourth (*D. scapigera*) nested inside Clade B. The two accessions of *D. longipetala*—the only species with a disjunct distribution—are not resolved as monophyletic; both samples are from Argentina in the same part of its range, however.

The full plastome ML phylogeny presents a substantially different set of relationships within *Deuterocohnia* and its relatives (Figure 3C). First, in the plastome tree *Deuterocohnia* is paraphyletic, with the single *Dyckia* sampled sister to plastome Clade 1 consisting of five species (*D. brevispicata*, *D. gableana*, *D. meziana*, *D. sanctae‐crucis*, *D. seramisiana*) that have branches of nearly zero length joining all subclades and the remaining species of *Deuterocohnia* forming plastome Clade 2 (Figure 3C). Plastome Clade 2 is also marked by several very short branches but contains two unusually long branches for *D. strobilifera* and *D. brevifolia*.

*Brocchinia* shows congruence of nuclear and plastome phylogenies, but there is extensive cytonuclear conflict between the nuclear and plastome trees in *Deuterocohnia* and *Pitcairnia*, with the conflict being somewhat greater in *Deuterocohnia* (Figure 3A, C). Clade F (*D. meziana*, *D. seramisiana*, *D. brevispicata*) is sister to the rest of *Deuterocohnia* in the nuclear tree, but its species are joined by *D. sanctae‐crucis*, *D. gableana*, and *Dyckia ibiramensis* in forming Clade 1 sister to the rest of the genus in the plastome tree.

Our tree simulation analysis indicates hybridization/introgression appears to be the likely cause of most cytonuclear conflict between the nuclear and plastome trees in *Deuterocohnia* and other Pitcairnioideae (Appendices S7 and S8). As explained in the Methods section, the low support scores within *Deuterocohnia* and *Pitcairnia* indicate that cytonuclear conflict is more likely to be explained by gene flow caused by hybridization/introgression and resulting plastome capture, rather than ILS (Appendices S7 and S8). Our network analysis confirms this view and suggests that relationships among species can best be explained by (1) a single gene‐flow event from *D. sanctae‐crucis* into the ancestor of nuclear Clade F with gamma = 0.254 (percentage of genetic exchange), or less likely (2) two gene‐flow events, including the first just mentioned (with gamma = 0.253), and a second *from D. brevispicata* into *D. seramisiana* with gamma = 0.486 (Appendices S9 and S10). There is a sharp decline in network pseudolikelihood scores in moving from hmax = 0 to 1 and then much smaller declines, suggesting that the true number of reticulations is equal to one. A survey of individual nuclear gene trees indicated that only 2.6% had one or more *Dyckia* species embedded in *Deuterocohnia*, and only 0.12% had all *Dyckia* species sampled embedded in *Deuterocohnia*.

*Deuterocohnia* has a crown age of 5.6 ± 1.4 Mya in the time‐calibrated ML‐constrained nuclear tree (Figure 4). *Deuterocohnia *+ *Dyckia* in the Bayesian full plastome phylogeny has a crown age of 8.7 ± 3.1 Mya; this same clade in the nuclear tree (in which *Deuterocohnia* is monophyletic) has a crown age of 8.5 ± 2.3 Mya (Figure 4). The crown age of subfamily Pitcairnioideae is 12.5 ± 2.9 Mya in the ML‐constrained nuclear tree (Figures 4) and 12.7 ± 3.7 Mya in the Bayesian full plastome tree (Appendices S11 and S12). The apparent age of chloroplast capture involving *Dyckia* and Clade 1 of *Deuterocohnia* is 4.4 ± 2.0 Mya and 6.7 ± 3.4 Mya based on the full plastome and plastid exon trees, respectively.

### Trait evolution in response to elevation and phylogeny in *Deuterocohnia*

**Figure 5:**
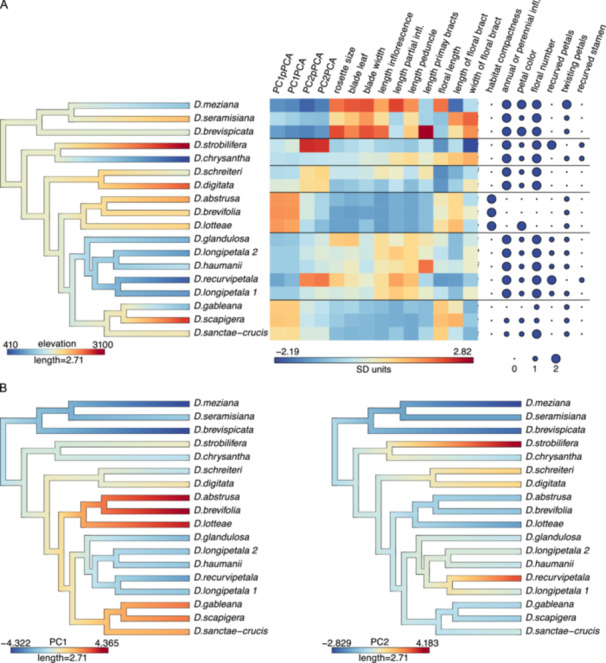
(A) Current and ancestral mean elevations mapped onto the nuclear BEAST phylogeny for *Deuterocohnia*. The adjacent heatmap indicates the standardized values of each trait and PCA scores. Categorical traits are plotted with dot maps. (B) Overlay of PC1 and PC2 from PCA; PC1 scores were reversed to correspond to the color scheme for elevation in panel A.

**Figure 6:**
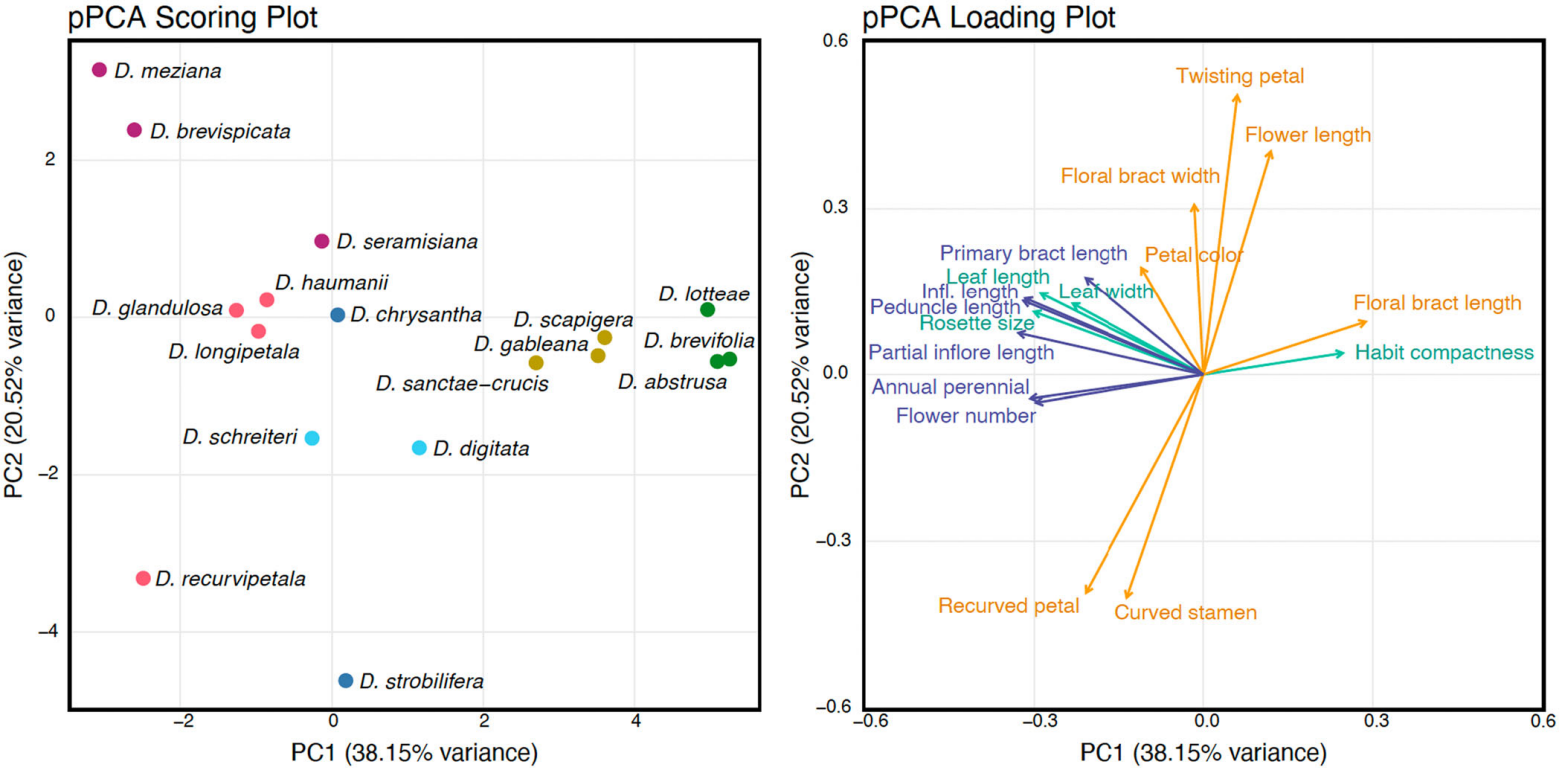
Phylogenetically structured PCA plots for *Deuterocohnia* species. Percentages of variance explained by PC1 and PC2 are indicated in axis labels. Left: Colors indicate each of six subclades within *Deuterocohnia*. Right: Color indicates the kinds of traits (yellow = floral traits, green = vegetative traits, blue = inflorescence traits).

Phylogenetically structured regressions show that rosette size (partial *r* ^*2*^ = 0.15, *P* = 0.04), inflorescence length (partial *r* ^*2*^ = 0.25, *P* = 0.02), partial inflorescence length (partial *r* ^*2*^ = 0.42, *P* = 0.006), and peduncle length (partial *r* ^*2*^ = 0.45, *P* < 0.001) show a significant decline with elevation in *Deuterocohnia* (Table 3). Flower length increases with increasing average temperature (partial *r* ^*2*^ = 0.33, *P* = 0.02). *Deuterocohnia chrysantha*—growing at low elevations but close to the cold Humboldt Current—is an exception to some of these trends, bearing leaves and rosettes of medium size. High‐elevation species also exhibit a more compact growth form and fewer flowers (Figure 5A). In the phylogenetically structured PCA (pPCA), PC1 explained 38.2% of the variance, while PC2 accounted for 20.5% (Figure 6). Vegetative and inflorescence traits are primarily explained by PC1, while floral traits are mostly captured by PC2 (Figure 6; Appendix S13). Additionally, PC1 scores from both PCA and pPCA show significant relationships with elevation (partial *r*² = 0.22, P = 0.04 for both PCA and pPCA). PC2 scores from pPCA are associated with mean annual temperature (partial *r*² = 0.33, *P* = 0.04), while PC2 scores from regular PCA show a marginally significant relationship (partial *r*² = 0.29, *P* = 0.07). The correlation of PC1 with elevation but not mean temperature likely reflects the cool (and dry) conditions at low elevations for *D. chrysantha* growing in the Atacama Desert, with the cold Humboldt Current offshore reducing lowland temperatures and unmeasured fog deposition replacing rainfall.

**Table 3:** Relationship between quantitative traits and environmental factors, including elevation (elev), precipitation (prec), and temperature (temp). PC1 and PC2 from both pPCA and PCA were extracted as trait variables. The best‐fitting model is shown; “*R*² total” indicates total variance explained, and “*R*² partial” is the variance explained by the major variable highlighted below (elev, prec, or temp). Adjusted *P*‐values with Yekutieli–Benjamini–Hochberg procedure (BH‐P) for each fixed effect are provided (*P < 0.05; **P < 0.01; ***P < 0.001). Sign of the slope indicates whether the trait variable increases (+) or decreases (−) with a unit increase in the environmental variables.

Formula	Best model	*R* ^2^ total	*R* ^2^ partial (variable)	BH‐P_elev_ (sign of slope)	BH‐P_prep_ (sign of slope)	BH‐P_temp_ (sign of slope)
Rosette size	~ elev + prec + P	0.57	0.15/0.1 (elev/prec)	0.04* (–)	0.07 (+)	—
Flower length	~ temp + NP	0.33	0.33 (temp)	—	—	0.02* (+)
Floral bract length	~ 1 + NP	‐‐	—	—	—	—
Floral bract width	~ 1 + P	0.07	—	—	—	—
Leaf length	~ temp + P	0.54	0.20 (temp)	—	—	0.04* (+)
Leaf width	~ 1 + P	0.56	—	—	—	—
Inflorescence length	~ elev + P	0.54	0.25 (elev)	0.02* (–)	—	—
Partial inflorescence length	~ elev + P	0.45	0.42 (elev)	0.006** (–)	—	—
Primary bract length	~ 1 + P	0.08	—	—	—	—
Peduncle length	~ elev + P	0.65	0.45 (elev)	0.0004*** (–)	—	—
PC1pPCA	~ elev + P	0.47	0.22 (elev)	0.04* (+)	—	—
PC2pPCA	~ temp + P	0.33	0.30 (temp)	—	—	0.04* (–)
PC1PCA	~ elev + P	0.50	0.22 (elev)	0.04* (–)	—	‐‐
PC2PCA	~ temp + P	0.29	0.24 (temp)	—	—	0.07 (+)

There is no clear tendency for mean elevation to evolve systematically upward or downward through time in *Deuterocohnia*; five invasions of higher elevations and four of lower elevations appear evident (Figure 5A). Clade F is sister to all other members of the genus and consists of three species found at low to intermediate elevations (200–2200 m a.s.l. in *D. meziana*; 1200–2200 m in *D. brevispicata*; and 2000–2400 m in *D. seramisiana* [Schütz, 2013]). The next divergent lineage—*D. chrysantha* and *D. strobilifera*—contains the two species with the lowest and highest elevational ranges, respectively (Figure 5A). The highest maximum elevations (>2600 m) evolved independently in four clades: B (*D. scapigera*), C (*D. abstrusa*, *D. brevifolia*, *D. lotteae*), D (*D. digitata*), and E (*D. strobilifera*) (Schütz, 2013; Figure 4). All these high‐elevation plants have evolved the cushion‐shrub growth form, with the most compact canopy surfaces in the three species from clade D, comprising three of the four species formerly segregated as *Abromeitiella*.

Closely related species in *Deuterocohnia* generally cluster together morphologically, as seen in the pPCA, except for *D. chrysantha* at low elevations along the Chilean coast and *D. strobilifera*, which reaches the highest elevation of any species in the genus (Figure 6). Although these two species are closely related, they are morphologically divergent. Both species have PC1 scores and morphological traits which, taken as a whole, place them with species from the middle of the elevational gradient (Figure 6). *Deuterocohnia strobilifera* has a high PC2 score, as does *D. recurvipetala* from low elevations (Figure 6); both species have evolved recurved petals and stamens.

### Historical biogeography and habitat shifts

**Figure 7:**
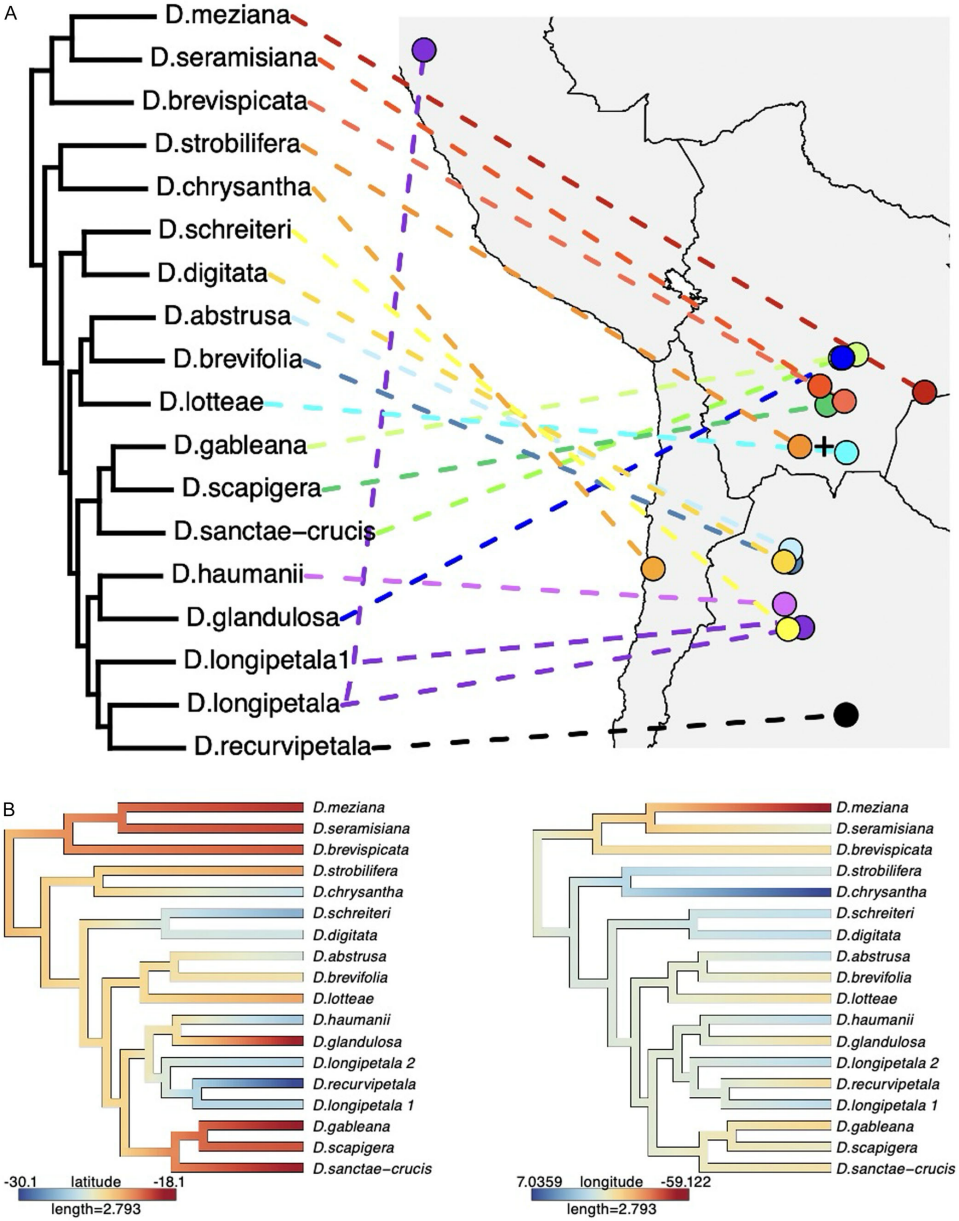
(A) Relationship between *Deuterocohnia* species relationships in the nuclear phylogeny and centroids (dots) of their distributional polygons. (B) ML overlay of ancestral mean latitude and longitude on the nuclear tree. Note the general progression to the south. Cross indicates inferred area of origin of *Deuterocohnia* at approximately 21.9° S, 64.8° W.

There was a general movement southward from an origin in south‐central Bolivia, based on a mapping of the distributional centroids of species onto the *Deuterocohnia* nuclear phylogeny (Figure 7). This pattern is punctuated by dispersal of Clade B (*D. gableana*, *D. sanctae‐crucis*, *D. scapigera*) and *D. glandulosa* to the north; of *D. chrysantha* from the central Andes to the Atacama Desert along the Chilean coast, and of *D. longipetala* from the central Andes into northern Peru. The inferred origin of the genus is roughly 21.9° S, 64.8° W (Figure 7).

**Figure 8:**
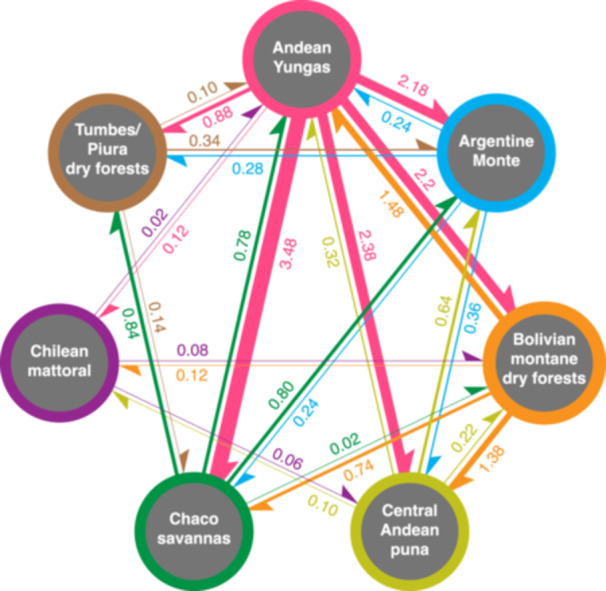
Mean number of dispersal events between pairs of habitats inferred from stochastic mapping of DEC model in BioGeoBears. Numbers and widths of arrows reflect the average number of dispersal events from source to target habitats. Andean Yungas is notable as the principal source from which divergence into different habitats occurs (see text).

DEC analyses in BioGeoBears identified the ancestral habitats of *Deuterocohnia* as being various combinations of Andean Yungas, Bolivian montane dry forests, central Andean puna, and Chilean matoral; except for the puna, these are found on mid‐elevation slopes of the central and southern Andes (Appendices S14 and S15). Stochastic mapping clearly identifies mid‐elevation Andean Yungas as the source area for most habitat diversification (Figure 8), with the major recipient habitats being chaco savannas, Argentine monte, Bolivian montane dry forests, and central Andean puna. The Andean Yungas are a humid subtropical region on the eastern slopes of the Andes with rugged terrain midway between rain forests and the Andean puna; the vegetation includes montane rain forests, cloud forests, and extensive areas of rocky outcrops. Most *Deuterocohnia* in the Andean Yungas and the drier Bolivian montane dry forests—and indeed, elsewhere—grow on exposed rocks (Schütz, 2013). Note that Bolivian montane dry forests are the second leading source for habitat diversification, contributing the only substantial input into the Andean Yungas and the second greatest input to the central Andean puna (Figure 8).

## DISCUSSION

### Target capture and sequencing

Our Bromeliad1815 bait kit effectively captured large numbers of single‐ or low‐copy nuclear genes across six bromeliad subfamilies, producing fully resolved, strongly supported, but discordant nuclear and plastome phylogenies for all 17 species of *Deuterocohnia* and 19 species of *Pitcairnia*. Both genera are resolved as monophyletic by the nuclear data; *Dyckia* is sister to *Deuterocohnia* in the nuclear trees and embedded within *Deuterocohnia* in the plastome trees. Tree simulation studies and network analyses suggest that cytonuclear conflict between the nuclear and plastome trees for *Deuterocohnia* partly reflects hybridization/introgression, with gene flow from *D. sanctae‐crucis* into the ancestor of nuclear Clade F, and possibly *from D. brevispicata* into *D. seramisiana* (Appendices [Link], [Link]). The sister relationships of monophyletic *Dyckia* and *Deuterocohnia* to each other in our nuclear phylogeny is consistent with previous studies based on the single‐copy genes PHYC and PRK (Schütz, 2013), which showed that *Deuterocohnia*, *Dyckia‐Encholirium*, and *Deuterocohnia‐Dyckia‐Encholirium* were monophyletic, with 99, 100, and 100% bootstrap support, respectively, and found almost no strong support for other interspecific relationships.

Target capture allows large numbers of low‐copy nuclear genes to be sequenced from dried herbarium material in many groups (e.g., Baker et al., 2022), but we had limited success doing so. Only 31% of herbarium samples yielded substantial numbers of nuclear gene sequences, and even they produced far fewer nuclear genes than fresh or silica‐dried material. Historically, it has been challenging to extract DNA even from such material in bromeliads (e.g., see Givnish et al., 1997), and virtually impossible to extract intact DNA from herbarium material and obtain sequences through PCR (G. Zizka, K. Weising, N. Schütz, personal observations). It is not clear why. We suggest that the abundant water‐storage tissue in the leaves of many bromeliads (especially in CAM groups with succulent foliage, like *Deuterocohnia*, *Dyckia*, *Encholirium*, and *Puya*) is the problem. Such hydrenchyma and thick leaves greatly slow the drying of leaf tissue and allow extensive fragmentation and degradation of DNA as cell compartments rupture during drying. DNA extraction from air‐dried photosynthetic tissue of other succulent plants is known to be difficult (e.g., Fehlberg et al., 2013), with slow drying contributing to DNA degradation, combined with other factors specific to those plants (e.g., high content of polysaccharides and defense compounds). The fact that nearly one‐third of the herbarium samples we processed yielded usable sequences via target capture, however, is a major step forward in obtaining usable DNA sequences from bromeliad herbarium specimens. This approach deserves further investigation.

### Phylogenetic reticulation

Reticulate evolution due to interspecific gene flow seems likely in *Deuterocohnia*, given the overlapping ranges of several species in a small area in southern Bolivia and northern Argentina, as well as the limited genetic divergence between species. The overlapping ranges of *Deuterocohnia* and *Dyckia* in that region also make intergeneric hybridization possible, but cytonuclear conflict indicates such hybridization occurred 4.4 Mya and had little persistent effect on the nuclear genome, affecting only 0.12–2.62% of the individual nuclear gene trees (see Results). By contrast, the species sampled in *Pitcairnia* occupy much more widely scattered ranges and have undergone greater genetic divergence from each other. The most likely patterns of interspecific gene flow—from *D. sanctae‐crucis* to the ancestor of the *brevispicata‐meziana‐seramisiana* clade in the network analysis—seem quite plausible, given that all four species have overlapping ranges in south‐central Bolivia (Figure 2) and all occur within a broad range of elevations (1200–2200 m) except *D. scapigera*, whose lower elevational limit is 2400 m but overlaps the elevational range of *D. sanctae‐crucis* (see data of Schütz, 2013).

### Phylogeny and historical biogeography

Our nuclear phylogeny implies an origin of *Deuterocohnia* in south‐central Bolivia, consistent with the hypothesis advanced by Givnish et al. (2011) based on the overlapping ranges there of the closely related genera *Fosterella*, *Deuterocohnia*, and *Dyckia* of Pitcairnioideae. South‐central Bolivia specifically seems likely as the birthplace of *Deuterocohnia*, given that area is where Clades F and A–E overlap, given the ancestral latitude and longitude reconstructed by our analyses and given that intermediate elevations are the ancestral state for the genus (see Figures 5A and 7). Clade F also appears to have originated in a small area of south‐central Bolivia (see distributions of *Deuterocohnia brevispicata* and *D. seramisiana* in Figure 2B, E) then spread into lower elevations in nearby parts of Brazil and Paraguay in *D. meziana*, which is the only species of the genus in those lowland areas (Figure 2A). The calculated point of origin of *Deuterocohnia* is just south of the Rio Pilcomayo, though so close (ca. 120 km) that not much should be made of that fact. Based on the nuclear tree, however, there appear to have been three dispersal events north of the Pilcomayo—Clade F, Clade B, and *D. glandulosa* in Clade A. On the other hand, the plastome tree is consistent with an origin north of the Rio Pilcomayo, and one dispersal event south of it, in Clade 2 minus *D. glandulosa*, which is sister to all other members of Clade 2.

The nuclear tree implies that *D. meziana*, *D. brevispicata*, and *D. seramisiana* are sister to all other *Deuterocohnia*, followed by *D. chrysantha*‐*D*. *strobilifera*. Schütz (2013) noted that morphological similarities unite these species, and Horres (2003) and Blank (2010) showed that they form a clade based on AFLP variation. The plastid and nuclear gene trees derived by Schütz (2013) and Schütz et al. (2016) were generally weakly resolved and supported and so produced few clear insights into relationships among species of *Deuterocohnia*. Exceptions included strong support for the monophyly of *Deuterocohnia*, *Dyckia‐Encholirium*, and *Deuterocohnia‐Dyckia‐Encholirium* in Schütz's (2013) nuclear tree, and for embedding of a monophyletic *Dyckia‐Encholirum* in paraphyletic *Deuterocohnia* in the plastome trees of Schütz (2013) and Schütz et al. (2016). Note that the nuclear tree of Schütz et al. (2016) placed *Dyckia‐Encholirium* sister to *Puya* with full support and *Deuterocohnia* sister to *Fosterella* with weak support. Our current study brings 1338 times as many aligned nuclear bases and 48 times as many aligned plastid bases as the pioneering study by Schütz (2013) 12 years ago and has, as expected, far greater resolution and support.

Based on a synthesis of morphological variation, biogeographic distributions, and phylogenetic analyses based on three plastid loci and two single‐copy nuclear loci, Schütz (2013) hypothesized six clades within *Deuterocohnia*, consisting of 1–6 species, with ancestral morphological characters like those of *D. longipetala*. With two exceptions, these clades do not match our nuclear or plastome clades. The first is the *meziana‐brevispicata‐seramisiana* clade, sister to all other *Deuterocohnia* in our nuclear tree and consisting of clade I envisioned by Schütz (2013). The second consists of subclade B of Schütz (2013), based on the ML analysis of three plastid loci, which consists of *D. meziana*, *D. brevispicata*, *D. seramisiana*, *D. scapigera*, and *D. gableana*. Subclade B is sister to *Dyckia* in the plastome analysis of Schütz (2013) and Schütz et al. (2016); the same species (except for *D. scapigera*, which did not sequence) are resolved as our plastome Clade 1 and sister to *Dyckia* in our plastome tree as well (Figures 3A and 4).

Schütz (2013) made the acute observation that all members of subclade B (our plastome Clade 1) lie north and east of the Rio Pilcomayo in south‐central Bolivia, while all members of her subclade A (our plastome Clade 2 and consisting of all other species in the genus) lie south and west of the Rio Pilcomayo. The sole exception to this rule is *D. glandulosa*, which lies south and west of the Pilcomayo and is sister to all other members of our plastome Clade 1). Schütz (2013) argued that the Rio Pilcomayo could have been a potent barrier to dispersal and perhaps especially during interglacial periods when swollen with meltwater. The fact that members of three nuclear clades (A, B, F) are found north of the Rio Pilcomayo, but all of those comprise plastome Clade 2 plus *D. glandulosa*, sister to all other members of plastome Clade 2, suggests that multiple dispersal events north of the Pilcomayo plus plastid capture by neighboring species there was responsible for the biogeographic pattern recognized by Schütz (2013). Our data support such hybridization and subsequent plastid capture between nuclear clade B and F ca. 4.3 Mya—long after *Deuterocohnia* arose and at a time when global temperature oscillations were much smaller than those of the Pleistocene 1.7 My later—but do not identify any reticulation involving *D. glandulosa* in nuclear clade A (Appendices 9 and 10). Schütz further hypothesized that the evolution of orange flowers in some members of subclade B might have facilitated hybridization with *Dyckia*, given the predominance of orange flowers in that genus. We note that hybridization with *Dyckia* in southeastern Bolivia, north of the Rio Pilcomayo, might also have caused the appearance of orange flowers in some subclade B/plastome Clade 1 species (i.e., *D. meziana* and *D. lotteae*).

*Deuterocohnia* includes four species previously classified in the genus *Abromeitiella*, i.e., *D. abstrusa* (part of former *A. lorentziana*), *D. brevifolia* (part of former *A. lorentziana*), *D. lotteae*, and *D. scapigera*. These species are characterized by a cushion‐shrub habit, short leaves, and one to a few greenish flowers on simple, short inflorescence (Figure 1; Schütz, 2013). Our nuclear target‐capture data confirm that three former *Abromeitiella* species—*D. abstrusa*, *D. brevifolia*, *D. lotteae*—form a clade embedded in *Deuterocohnia*. The fourth species—*D. scapigera*, which has a less compact growth form—is instead closely related to *D. gableana* and *D. sanctae‐crucis* based on nuclear sequence data. Spencer and Smith (1992) synonymized *Abromeitiella* into *Deuterocohnia* due to their overwhelming similarity in several morphological traits (e.g., asymmetric sepals, wholly superior ovary, comma‐shaped appendaged seeds) and the presence of short scapes and cushion‐like habit in two *Deuterocohnia* species (*D. digitata*, *D. strobilifera*). The latter species are like each other and diverge from other high‐elevation cushion‐forming species in having somewhat longer and incurved leaves, forming somewhat “rougher” cushions at high elevations.

### Nuclear vs. plastid phylogenies

We note that the rate of nucleotide substitution and resulting branch lengths in the nuclear phylogeny are several times greater than those in the plastome phylogeny (Figure 3C). This is consistent with the expectation that, other things being equal, genetic drift should result in substitution rates four times greater in nuclear vs. plastid sequences (e.g., see Drouin et al., 2008). The much greater rate of genetic divergence probably also underlies the lower support for several nodes in the plastome vs. nuclear phylogeny. The greater amount of genetic divergence in the nuclear data, the greater support for relationships in the nuclear phylogeny, and the greater vulnerability of the plastome phylogeny to distortions caused by chloroplast capture associated with hybridization and/or introgression all argue for giving primacy to the nuclear phylogeny—especially the ASTRAL tree—in assessing evolutionary relationships among *Deuterocohnia* species. In *Deuterocohnia*, the ASTRAL nuclear tree and ML nuclear tree have the same branching topology and thus support the same relationships among species (Figure 3C).

The strong support provided by nuclear data for the reciprocal monophyly of *Deuterocohnia* and *Dyckia*, and the evidence that the past hybridization between ancestral taxa that led to chloroplast capture from *Deuterocohnia* by *Dyckia* has left few traces in the nuclear genome, provides conclusive evidence to reject the transfer of *Deuterocohnia* species to *Dyckia* by Gomes‐Da‐Silva et al. (2019).

Our nuclear ML and ASTRAL phylogenies based on the Bromeliad1815 bait kit confirm that relationships among the six subfamilies sampled are congruent with those inferred from DNA sequences for eight plastid loci (Givnish et al., 2011, 2014). Our findings contrast with those of Yardeni et al. (2022) based on the original Bromeliad1776 kit, which placed Hechtioideae sister to Tillandsioideae instead of the clade (Pitcairnioideae, (Puyoideae, Bromelioideae)); placed *Deuterocohnia* sister to *Puya* instead of *Pitcairnia*; and failed to resolve Pitcairnioideae as monophyletic. All of these are inconsistent with the plastid phylogeny of Givnish et al. (2011) and nuclear phylogenies in this paper, which are consistent with each other. These differences suggest that our Bromeliads1815 kit generates more reliable nuclear data than Bromeliads1776 for reconstructing evolutionary relationships across Bromeliaceae and within Pitcairnioideae. Differences in taxon sampling between our study and that of Yardeni et al. (2022) might also contribute to the differences observed, but the substantial documented divergence of many baits in Bromeliads1776 from genomes representing several bromeliad subfamilies—and our inclusion of additional baits in Bromeliads1815 to target sequences in other subfamilies—provide a plausible explanation for the differences in phylogenies reconstructed.

### Morphological and ecological trends in *Deuterocohnia*

*Deuterocohnia* exhibits a trend toward increasing compactness and reductions in leaf size, rosette diameter, inflorescence length, and number of flowers per inflorescence with elevation (Figure 5A; Appendices 11 and 12). This morphological gradient likely reflects an adaptive response to environmental pressures associated with higher elevations, including increased wind exposure, decreased rainfall, and lower temperatures that can reduce root function. These conditions can favor smaller leaves (Givnish, 1979) and developmentally correlated reductions in rosette and inflorescence size, and favor more compact cushion‐shrub growth forms to minimize water loss and mechanical damage from winds and snow and increase leaf temperatures (Arroyo et al., 2003; Körner, 2003; Badano et al., 2006; Michalet et al., 2014; Givnish, 1979, 2016). Smaller leaves can also facilitate the ability of plants to form the aerodynamically smooth canopies of cushion shrubs. Similar adaptive trends have been observed in other alpine and montane plants, where increased compactness (Milla et al., 2008) and reduced leaf surface area (Zhang et al., 2020) are associated with elevated altitudes.

Ecological niche conservatism is evident within *Deuterocohnia*, with morphologically and elevationally similar species forming clades, based on our nuclear phylogeny and PCA and pPCA of morphological traits. Nevertheless, as noted above, morphological variation does not imply the relationships documented by rapidly evolving target‐capture nuclear sequences. Furthermore, two closely related species deviate greatly in elevation: *D. chrysantha*, endemic to the Atacama Desert, occupies the lowest elevation range, while *D. strobilifera* reaches alpine regions up to 4000 m, the highest for the genus. *Deuterocohnia chrysantha*, native to one of the driest deserts with 0–50 mm annual precipitation (but with unmeasured amounts of fog deposition), displays larger, less‐compact rosettes and longer leaves, perhaps for heat dissipation (Schütz, 2013) or instead reflecting greater productivity under cool, misty conditions near the cold Humboldt Current offshore than under cold, dry, windy low‐humidity conditions at high elevations in the Andes. Under such conditions, *D. strobilifera* has evolved a low stature, small leaves, rosettes, and inflorescences, and an aerodynamically smooth cushion‐shrub form, crucial for surviving the harsh alpine environment. This rapid morphological divergence between *D. chrysantha* and *D. strobilifera* underscores the rapid selective impact of adaptation to extreme habitats. The acquisition of the same suite of traits by six species in four clades of *Deuterocohnia* is prima facie evidence—via convergent evolution—of the adaptive value of such traits in high‐elevation Andean environments. Notably, individual flower traits such as floral bract length, flower length, and recurved stamens during anthesis show little to no response to elevation gradients. Perhaps these traits are influenced by selective pressures related to pollinator availability and activity.

Our analyses suggest that the Andean Yungas at intermediate elevations is the leading source for dispersal/evolution into other major habitats, including lower‐elevation savannas of the semi‐arid Gran Chaco in southern Bolivia, western Paraguay, and northern Argentina; the treeless puna at high elevations in the Central Andes; Bolivian montane dry forests at intermediate elevations on the eastern side of the Andes; and the arid lowland thorn scrub and grassland of the Argentine Monte (see above). Bolivian montane dry forests are the second leading for such dispersal/evolution into other habitats and are ecologically intermediate between puna and chaco scrub. These forests and the Yungas are often found in steep, landslide‐prone areas of high tectonic activity, with deep valleys and high ridges in close proximity, and with extensive areas of exposed rocks locally and in communities up‐ and downslope. These conditions favor dispersal by *Deuterocohnia* into other rocky habitats at higher and lower elevations nearby. Open rocky terrain favors *Deuterocohnia* because many species are quite short in stature, and are shade‐intolerant, drought‐adapted, and have slow growth rates coupled to possession of CAM photosynthesis. Terrestrial *Deuterocohnia* is thus likely to persist only on thin soils on open sites and unlikely to compete successfully on deep soils under moist conditions. It is thus not surprising that many *Deuterocohnia* species are especially common in these mid‐slope communities on unstable slopes and on dry sites at low and high elevations nearby.

In the immediate future, the current study should be expanded to include multiple accessions per species within *Deuterocohnia* to test the monophyly of each, especially of *D. longipetala* with its disjunct range. The lack of monophyly of several species even in the Bayesian analysis of plastid sequences by Schütz (2013) makes such research a high priority. Our nuclear phylogeny for *Deuterocohnia* opens the door for reconstructing trait evolution across species. In addition, companion studies should be conducted to study relationships across the much larger and wider *Dyckia‐Encholirium* complex and *Pitcairnia* should be conducted using approaches as or more powerful than those used here. Such studies would permit analyses of trait evolution, taxonomic delimitation, and the role of hybridization/introgression and ecological divergence on diversification of the entire subfamily Pitcairnioideae.

## AUTHOR CONTRIBUTIONS

B.L. collected and extracted DNA for a few accessions, designed the Bromeliad1815 bait kit, conducted all phylogenetic, historical biogeographic, and statistical analyses, and wrote the first draft of the manuscript. N.S. collected most of the accessions in the field, extracted DNA, and contributed many useful comments. K.W. and G.Z. supervised phylogenetic research by N.S. and provided helpful comments. J.B.L. helped design the Bromeliad1815 bait kit and provided advice on some analyses. T.J.G. conceived the project, revised the manuscript, and provided funding. All authors reviewed the revised paper, suggested additional edits, and approved the final draft.

## Supporting information


**Appendix S1**. Links for draft genomes at CoGe.

**Appendix S2**. Lists of changes made in moving from the Bromeliad1776 bait kit to the Bromeliad1815 bait kit.

**Appendix S3**. Table of accessions and assembly data.

**Appendix S4**. Additional information about genome assembly.

**Appendix S5**. Comparison of BioGeoBears models used to estimate habitat evolution in *Deuterocohnia*.

**Appendix S6**. Gene concordance factors (gCFs) on maximum likelihood nuclear tree.

**Appendix S7**. The percentage of simulated trees under MSC supporting specific clades on the BEAST full plastome tree.

**Appendix S8**. The percentage of simulated trees under MSC supporting specific clades on the ML full plastome tree.

**Appendix S9**. Comparison of pseudolikelihood scores for networks generated by SnaQ.

**Appendix S10**. SnaQ results.

**Appendix S11**. Bayesian tree reconstructed from BEAST using full plastome data set.

**Appendix S12**. Bayesian tree reconstructed from BEAST using plastid exon data set.

**Appendix S13**. Phylogenetically structured and regular PCA (bottom) based on morphological traits.

**Appendix S14**. Most likely ancestral habitats at each node under DEC as implemented in BioGeoBears.

**Appendix S15**. Distribution of likely ancestral habitats at each node under DEC.

## Data Availability

The sequencing data generated during this study have been deposited in the Sequence Read Archive (SRA) under project ID: PRJNA1184165. The data set is accessible at https://www.ncbi.nlm.nih.gov/bioproject/?term=PRJNA1184165. The alignment files and tree files for nuclear, full plastome, and plastid exon are deposited at Figshare: https://figshare.com/s/55d31ed6c7258b6a22b4. Bait sequences are also available at Figshare: https://doi.org/10.6084/m9.figshare.30472904; the baits themselves can be obtained from Daicel Arbor. Genome sequences are deposited in CoGe (see Materials and Methods for links).
