# Supplementary material for: Phylogenomics, ecomorphological evolution, and historical biogeography in Deuterocohnia (Bromeliaceae: Pitcairnioideae)
Source: Am J Bot. 2026 Jan 28;113(2):e70153. doi: 10.1002/ajb2.70153 (PMC12918849; doi:10.1002/ajb2.70153)
Supplement: Supplementary file 1 — Appendix S1. Links for draft genomes at CoGe. [file AJB2-113-e70153-s002.docx]

**Appendix S1**. Links for draft genomes at CoGe.

| **Species** | **Name** | **Link** |
| --- | --- | --- |
| *Pitcairnia atrorubens* | Pit_atr_primary.genome.scf.fasta | <https://genomevolution.org/r/948hv> |
| *Navia splendens* | Nav_spl_primary.genome.scf.fasta | <https://genomevolution.org/r/948hs> |
| *Lindmania longipes* | Lin_sal_primary.genome.scf.fasta | <https://genomevolution.org/r/948ho> |
| *Hechtia lundelliorum* | Hec_lun_primary.genome.scf.fasta | <https://genomevolution.org/r/948hk> |
| *Brocchinia reducta* | Bro_red_primary.genome.scf.fasta | <https://genomevolution.org/r/948hd> |
| *Brocchinia paniculata* | Bro_pan_primary.genome.scf.fasta | <https://genomevolution.org/r/948gz> |
| *Brocchinia acuminata* | Bro_acu_primary.genome.scf.fasta | <https://genomevolution.org/r/948h8> |
