# Supplementary material for: Phylogenomics, ecomorphological evolution, and historical biogeography in Deuterocohnia (Bromeliaceae: Pitcairnioideae)
Source: Am J Bot. 2026 Jan 28;113(2):e70153. doi: 10.1002/ajb2.70153 (PMC12918849; doi:10.1002/ajb2.70153)
Supplement: Supplementary file 2 — Appendix S2. Lists of changes made in moving from the Bromeliad1776 bait kit to the Bromeliad1815 bait kit. [file AJB2-113-e70153-s005.docx]

**Appendix S2**. Lists of changes made in moving from the Bromeliad1776 bait kit to the Bromeliad1815 bait kit.

**List 1. 170 target genes from Bromeliad 1776 that were removed due to low resolution in tree building. Removing those genes resulted in 3113 baits removed from Bromeliad 1776 bait kit.**

Aco000319; Aco000482; Aco000722; Aco000985; Aco001473; Aco001647; Aco001848; Aco003507; Aco003826; Aco005055; Aco005359; Aco006594; Aco006847; Aco006923; Aco007119; Aco007509; Aco007828; Aco008685; Aco008947; Aco009301; Aco010827; Aco013178; Aco013390; Aco013718; Aco014855; Aco015377; Aco015589; Aco017324; Aco018949; Aco018982; Aco019058; Aco021447; Aco022851; Aco031044; Aco000736; Aco000748; Aco001003; Aco001430; Aco001542; Aco001571; Aco001826; Aco002037; Aco002227; Aco002366; Aco002400; Aco002616; Aco002683; Aco002887; Aco003190; Aco003430; Aco003654; Aco003688; Aco004375; Aco004393; Aco004555; Aco004773; Aco004861; Aco004946; Aco005083; Aco005262; Aco005826; Aco005868; Aco005975; Aco006030; Aco006056; Aco006252; Aco006416; Aco006438; Aco006536; Aco006540; Aco006586; Aco006719; Aco007198; Aco007294; Aco007319; Aco007629; Aco007670; Aco007730; Aco007832; Aco008330; Aco008684; Aco009346; Aco009385; Aco009469; Aco009514; Aco009549; Aco009551; Aco009599; Aco009600; Aco009627; Aco009903; Aco010086; Aco010160; Aco010298; Aco010312; Aco010341; Aco010438; Aco010619; Aco010748; Aco010779; Aco011075; Aco011346; Aco011515; Aco011705; Aco012179; Aco012412; Aco012577; Aco012793; Aco012797; Aco012825; Aco012927; Aco012941; Aco013015; Aco013038; Aco013080; Aco013204; Aco013309; Aco013510; Aco013642; Aco013744; Aco013769; Aco013938; Aco014063; Aco014077; Aco014120; Aco014342; Aco014555; Aco014579; Aco014837; Aco015194; Aco015317; Aco015319; Aco015349; Aco015443; Aco015521; Aco016261; Aco016352; Aco016876; Aco017006; Aco017527; Aco017705; Aco017802; Aco017874; Aco018101; Aco018176; Aco018265; Aco018269; Aco018321; Aco018512; Aco018517; Aco018533; Aco018578; Aco018637; Aco019116; Aco019362; Aco019534; Aco021008; Aco021616; Aco021721; Aco022384; Aco022696; Aco022927; Aco023000; Aco023573; Aco023600; Aco024340; Aco025623; Aco026485; Aco027040; Aco031276.

List 2. 209 locus variants added to the modified Bromeliad 1815 bait kit. The string attached to the locus ID shows which species this locus variant comes from.

Aco000385_Brored; Aco000403_Brored; Aco000425_Brored; Aco000456_Broacu; Aco000487_Broacu; Aco000551_Bropan; Aco000674_Brored; Aco000727_Bropan; Aco001437_Bropan; Aco001482_Brored; Aco001651_Bropan; Aco001742_Brored; Aco001853_Broacu; Aco001958_Bropan; Aco002475_Broacu; Aco002475_Navspl; Aco002508_Bropan; Aco002697_Broacu; Aco002739_Linsal; Aco002744_Broacu; Aco002744_Linsal; Aco003321_Bropan; Aco003456_Brored; Aco003857_Bropan; Aco003885_Broacu; Aco004290_Bropan; Aco004290_Brored; Aco004406_Bropan; Aco004672_Broacu; Aco004708_Bropan; Aco005168_Bropan; Aco005173_Bropan; Aco005229_Brored; Aco005239_Brored; Aco005287_Broacu; Aco005354_Bropan; Aco005358_Bropan; Aco005358_Linsal; Aco005373_Bropan; Aco005393_Brored; Aco005399_Bropan; Aco005402_Linsal; Aco005409_Broacu; Aco005444_Brored; Aco005478_Broacu; Aco005562_Linsal; Aco005567_Broacu; Aco005567_Navspl; Aco005587_Broacu; Aco005908_Bropan; Aco005908_Heclun; Aco005908_Navspl; Aco006063_Brored; Aco006064_Broacu; Aco006084_Bropan; Aco006467_Brored; Aco006487_Brored; Aco007041_Broacu; Aco007085_Brored; Aco007277_Broacu; Aco007277_Bropan; Aco007469_Broacu; Aco007490_Broacu; Aco007539_Bropan; Aco007580_Broacu; Aco007659_Brored; Aco008078_Brored; Aco008287_Broacu; Aco008315_Brored; Aco008429_Brored; Aco008510_Bropan; Aco008626_Brored; Aco008744_Brored; Aco008775_Linsal; Aco008882_Broacu; Aco008882_Navspl; Aco008925_Brored; Aco009272_Brored; Aco009353_Brored; Aco009371_Bropan; Aco009371_Navspl; Aco009559_Brored; Aco009569_Bropan; Aco009587_Pitatr; Aco009587_Broacu; Aco009612_Bropan; Aco009686_Bropan; Aco009686_Navspl; Aco009713_Broacu; Aco009889_Broacu; Aco010013_Bropan; Aco010034_Brored; Aco010251_Bropan; Aco010251_Linsal; Aco010367_Bropan; Aco010614_Pitatr; Aco010614_Broacu; Aco010614_Heclun; Aco010614_Linsal; Aco010641_Broacu; Aco010847_Broacu; Aco011011_Bropan; Aco011025_Bropan; Aco011051_Broacu; Aco011105_Broacu; Aco011198_Bropan; Aco011246_Broacu; Aco011700_Broacu; Aco011700_Bropan; Aco011709_Puyrai; Aco011903_Bropan; Aco012745_Bropan; Aco012745_Heclun; Aco012745_Linsal; Aco012745_Navspl; Aco012772_Broacu; Aco012772_Bropan; Aco012776_Broacu; Aco013121_Broacu; Aco013227_Pitatr; Aco013227_Broacu; Aco013227_Linsal; Aco013253_Broacu; Aco013355_Brored; Aco013474_Broacu; Aco013536_Brored; Aco013584_Navspl; Aco013643_Broacu; Aco013659_Brored; Aco013975_Brored; Aco013992_Brored; Aco014274_Broacu; Aco014404_Brored; Aco014415_Pitatr; Aco014474_Bropan; Aco014590_Linsal; Aco014693_Brored; Aco014753_Broacu; Aco014753_Bropan; Aco014755_Bropan; Aco014884_Bropan; Aco014942_Broacu; Aco015042_Broacu; Aco015141_Broacu; Aco015198_Broacu; Aco015199_Broacu; Aco015741_Brored; Aco015741_Navspl; Aco015799_Broacu; Aco016014_Brored; Aco016029_Brored; Aco016047_Broacu; Aco016151_Bropan; Aco016255_Brored; Aco016318_Bropan; Aco016390_Bropan; Aco016393_Brored; Aco016654_Broacu; Aco016710_Broacu; Aco016712_Broacu; Aco016732_Broacu; Aco016748_Pitatr; Aco016748_Bropan; Aco016748_Navspl; Aco016898_Bropan; Aco016948_Bropan; Aco017042_Broacu; Aco017128_Brored; Aco017128_Navspl; Aco017169_Brored; Aco017195_Bropan; Aco017624_Broacu; Aco017965_Brored; Aco017965_Linsal; Aco017967_Bropan; Aco018117_Brored; Aco018141_Broacu; Aco018141_Navspl; Aco018268_Brored; Aco018350_Bropan; Aco018674_Pitatr; Aco018674_Broacu; Aco019622_Broacu; Aco019932_Bropan; Aco020155_Broacu; Aco020162_Bropan; Aco020317_Brored; Aco020843_Puyrai; Aco020843_Broacu; Aco021318_Bropan; Aco021905_Broacu; Aco022074_Bropan; Aco022172_Brored; Aco022563_Broacu; Aco022847_Broacu; Aco022847_Linsal; Aco023089_Pitatr; Aco023089_Brored; Aco023089_Linsal; Aco023089_Navspl; Aco023190_Brored; Aco023190_Heclun; Aco024020_Brored; Aco024634_Broacu; Aco024749_Broacu; Aco026097_Brored; Aco026631_Broacu; Aco027726_Bropan; Aco030333_Pitatr.
