# Supplementary material for: Phylogenomics, ecomorphological evolution, and historical biogeography in Deuterocohnia (Bromeliaceae: Pitcairnioideae)
Source: Am J Bot. 2026 Jan 28;113(2):e70153. doi: 10.1002/ajb2.70153 (PMC12918849; doi:10.1002/ajb2.70153)
Supplement: Supplementary file 4 — Appendix S4. Additional information about genome assembly. [file AJB2-113-e70153-s003.docx]

**Appendix S4.** Additional information about genome assembly

**Additional *Deuterocohnia, Dyckia*, and *Fosterella* genomes from GenBank**

To identify potentially useful samples within subfamily Pitcairnioideae, we thoroughly searched GenBank and Sequence Read Archive (SRA) for the genera *Fosterella*, *Encholirium*, and *Dyckia*. In addition to two outgroup *Typha* species sequenced by whole-genome sequencing, we found two *Fosterella* species sequenced by Illumina RNA-seq and genome skimming, respectively, and four *Dyckia* species sequenced by Illumina RNA-seq. Listed below are sequences we found in SRA. Information about assembly and alignment quality is given in Appendix S1.

Here, we describe our approaches for analyzing different data types downloaded online:

- Whole-genome sequencing or genome-skimming data:

Two outgroup *Typha* species (SRR15691252, SRR15680749) were sequenced by Illumina whole-genome sequencing. *Deuterocohnia glandulosa* (SRR13700303) and *Fosterella penduliflora* (SRR13700302), sequenced by low-coverage genome skimming, were added as additional samples (SRA BioProject: [PRJNA701548](https://www.ncbi.nlm.nih.gov/bioproject/PRJNA701548)) (Appendix S1). Those samples went through the same procedures described in the article for data trimming, assembly, alignment and phylogenetic tree building. However, the two *Typha* samples has a large number of genes missing after assemblies (70% missing bases in the concatenated nuclear data set) because our target capture bait kit is designed for Bromeliaceae ingroup while *Typha* is distantly related. To ensure an outgroup outside Bromeliaceae, we included those two samples into our nuclear tree building. *Deuterocohnia glandulosa* and *F. penduliflora* failed the nuclear assembly process because of low coverage (Appendix S1), generating almost all missing bases in the nuclear data set. However, all samples here generated good assembly for full plastome and plastid exons and thus are included in our plastome phylogenies.

- Illumina RNA-seq sequencing

We downloaded four RNA-seq sequencing samples from SRA, including *Dyckia velascana* (SRR29188277), *Dyckia remotiflora* var. *montevidensis* (SRR2918827), *Dyckia niederleinii* (SRR29188275), and *Dyckia leptostachya* (SRR29188274). We used the same quality check and nuclear and plastome assembly approaches as described in the manuscript (see Methods). Those sample passed nuclear assembly while failing generating high quality plastome sequences, so they were only included in our nuclear tree. Assembly quality of those four samples is provided in Appendix S1.

- Nanopore long-read Dyckia ibiramensis (PRJNA872198)

We identified one relevant whole-genome sequencing project for Dyckia ibiramensis, sequenced using Nanopore MinION (PRJNA872198). This sample was processed with Porechop v. 0.2.4 (<https://github.com/rrwick/Porechop>) to remove adaptor sequences, followed by trimming reads shorter than 500 bp and with quality scores below 10 using NanoFilt (De Coster W, 2018). Single-copy nuclear genes were extracted from the cleaned long reads using Bowtie2 (Langmead and Salzberg 2012), SAMtools (Li et al. 2009), and BCFtools (Li 2011). However, after alignment and trimming, the sample exhibited 60% missing data, which was subsequently excluded from downstream analysis.

**Parameters of GetOrganelle**

To optimize parameters for GetOrganelle, we tested a data set consisting of six *Brocchinia* (001, 037, 038, 039, Bro_pan, 043), one *Puya* (Puy.rai), one *Pitcairnia* (Pit.atr), one *Hechtia* (Hec.lun), and six *Deuterocohnia* (018, 019, 020, 021, 022, 023) samples (Appendix S1). We evaluated different seed data sets and parameters, using Geneious and Bandage (Wick et al. 2015) to visually assess assembly quality. Increasing the default *k*-mer length to *k* = 127 and shortening the *k*-mer interval resulted in longer contigs and higher per-base assembly coverage. While using different seed data sets increased per-base coverage, it did not significantly enhance contig length or completeness. Adjusting word values manually did not improve assembly quality. Consequently, we chose the parameters -R 15 -k 21,35,45,65,75,85,105,127 with the plastome assembly of *Puya mirabilis* (NC 045380.1) as the seed data set. Low-coverage genome skimming samples generated full circular plastomes with 300–500*x* per-base coverage. However, samples sequenced using target capture sequencing often failed to retrieve complete plastomes, as expected due to the selective targeting of specific regions by the modified Bromeliad bait kit. For these samples, we re-ran GetOrganelle with parameters -R 20 -k 21,35,45,65,75,85,95,105,127, using genus-specific seed data sets. For instance, we included references such as *Brocchinia hechtioides* (NC069197.1), *Pitcairnia atrorubens* (NC085612.1), *Hechtia stenopetala* (NC080304.1), *Lindmania* sp*.* (OQ308827.1), and *Navia igneosicola* (NC085611.1). No *Deuterocohnia* reference plastome was available on GenBank, so we used *Pitcairnia atrorubens* as a proxy for *Deuterocohnia* samples. We included Dyckia ibiramensis (OR832762.1) from GenBank into phylogeny reconstruction but we did not use it as a reference because it was labelled as “unverified” in GenBank.

**Assemblies of plastid exon data set**

In addition to reference-guided de novo full plastome assemblies, we assembled plastid exons, referred to as the plastid exon data set. A full plastome assembly of *Puya mirabilis* (NC 045380.1) was downloaded from GenBank and annotated using Chole (https://chloe.plastid.org/). This reference was selected based on multiple tests using the test data set used in GetOrganelle testing. After removing introns, rRNA, tRNA, and genes from the second inverted repeat, we retained coding regions from the non-repeat and first inverted repeat regions as the reference for assembling plastid exons. For genes with multiple exons, exons were concatenated to represent each gene. We assembled plastid exons from all samples using Hybpiper, including the extracted exon regions from the reference as the target file. We also extracted plastid exons from online samples downloaded from GenBank, including *Brocchinia hechtioides* (NC069197.1), *Pitcairnia atrorubens* (NC085612.1), *Hechtia stenopetala* (NC080304.1), *Landmania sp.* (OQ308827.1), *Navia igneosicola* (NC085611.1), and *Dyckia plastome* (OR832762.1). The same procedure was applied to build maximum likelihood phylogenies using IQ-Tree2 and Bayesian phylogenies with MrBayes and BEAST.

**References**

Chen, S., Zhou, Y., Chen, Y., & Gu, J. (2018). fastp: an ultra-fast all-in-one FASTQ preprocessor. *Bioinformatics 34*(17), i884–i890. <https://doi.org/10.1093/bioinformatics/bty560>

De Coster W, D’Hert S, Schultz DT, Cruts M, Van Broeckhoven C. (2018) NanoPack: visualizing and processing long-read sequencing data. *Bioinformatics* 34: 2666–2669

Langmead B and Salzberg SL. (2012) Fast gapped-read alignment with Bowtie 2. *Nature Methods* 9: 357–359. doi: 10.1038/nmeth.1923.

Li H (2011) A statistical framework for SNP calling, mutation discovery, association mapping and population genetical parameter estimation from sequencing data. *Bioinformatics* 27: 2987–2993.

Li H, Handsaker B, Wysoker A, et al. (2009) The Sequence Alignment/Map format and SAMtools. *Bioinformatics* 25: 207–2079. doi:10.1093/bioinformatics/btp352.

Wick R, Schultz M, Zobel J, Holt K (2015) Bandage: interactive visualization of de novo genome assemblies. Bioinformatics 31(20): 3350–3352.
