# Supplementary material for: Phylogenomics, ecomorphological evolution, and historical biogeography in Deuterocohnia (Bromeliaceae: Pitcairnioideae)
Source: Am J Bot. 2026 Jan 28;113(2):e70153. doi: 10.1002/ajb2.70153 (PMC12918849; doi:10.1002/ajb2.70153)
Supplement: Supplementary file 5 — Appendix S5. Comparison of BioGeoBears models used to estimate habitat evolution in Deuterocohnia. [file AJB2-113-e70153-s012.docx]

**Appendix S5.** Comparison of BioGeoBears models used to estimate habitat evolution in *Deuterocohnia*. DEC is the preferred model, based on lowest log likelihood (tied with DEC + J), lowest AICc, and highest AICc weight. Parameters *d*, *e*, and *j* refer to the rate of dispersal or range expansion, local extinction or range contraction, and founder-event speciation, respectively.

**Model Ln L No.parameters *d e j* AICc AICc wgt**

DEC -65.94 2 0.045 1.0E-12 0 136.7 0.56

DEC+J -65.94 3 0.045 1.0E-12 0.0001 139.6 0.13

DIVALIKE -67.42 2 0.053 1.0E-12 0 139.6 0.13

DIVALIKE+J -67.32 3 0.051 1.0E-12 0.0059 142.4 0.033

BAYAREALIKE -67.49 2 0.031 0.19 0 139.8 0.12

BAYAREALIKE+J -67.49 3 0.031 0.19 1.0E-05 142.7 0.028
