# Supplementary material for: Phylogenomics, ecomorphological evolution, and historical biogeography in Deuterocohnia (Bromeliaceae: Pitcairnioideae)
Source: Am J Bot. 2026 Jan 28;113(2):e70153. doi: 10.1002/ajb2.70153 (PMC12918849; doi:10.1002/ajb2.70153)
Supplement: Supplementary file 6 — Appendix S6. Gene concordance factors (gCFs) on maximum likelihood nuclear tree. [file AJB2-113-e70153-s009.docx]

Appendix S6. Gene concordance factors (gCFs) on maximum likelihood nuclear tree.
