# Supplementary material for: Phylogenomics, ecomorphological evolution, and historical biogeography in Deuterocohnia (Bromeliaceae: Pitcairnioideae)
Source: Am J Bot. 2026 Jan 28;113(2):e70153. doi: 10.1002/ajb2.70153 (PMC12918849; doi:10.1002/ajb2.70153)
Supplement: Supplementary file 7 — Appendix S7. The percentage of simulated trees under MSC supporting specific clades on the BEAST full plastome tree. [file AJB2-113-e70153-s007.docx]

**Appendix S7.** Percentage of simulated trees under MSC supporting specific clades on BEAST full plastome tree. No percentage is strictly zero, and percentages were rounded to two decimal places. Only overlapping species between the Astral species tree and both ML and Bayesian full plastome trees are included, so *Dyckia* species were removed due to non-overlapping species.
