# Supplementary material for: Phylogenomics, ecomorphological evolution, and historical biogeography in Deuterocohnia (Bromeliaceae: Pitcairnioideae)
Source: Am J Bot. 2026 Jan 28;113(2):e70153. doi: 10.1002/ajb2.70153 (PMC12918849; doi:10.1002/ajb2.70153)
Supplement: Supplementary file 8 — Appendix S8. The percentage of simulated trees under MSC supporting specific clades on the ML full plastome tree. [file AJB2-113-e70153-s014.docx]

**Appendix S8.** The percentage of simulated trees under MSC supporting specific clades on the ML full plastome tree. No percentage is strictly zero and percentages were rounded to 2 decimal places. Only overlapping species between the Astral species tree and both ML and Bayesian full plastome trees are included, so *Dyckia* species are removed due to non-overlapping species.
