# Supplementary material for: Phylogenomics, ecomorphological evolution, and historical biogeography in Deuterocohnia (Bromeliaceae: Pitcairnioideae)
Source: Am J Bot. 2026 Jan 28;113(2):e70153. doi: 10.1002/ajb2.70153 (PMC12918849; doi:10.1002/ajb2.70153)

**Appendix S9.** Comparison of pseudolikelihood scores for networks generated by SNaQ. The *x*-axis represents Hmax, which indicates the number of reticulation events allowed for each network estimation, while the *y*-axis shows the pseudolikelihood scores. Each network was generated using 20 runs. The true number for Hmax should be indicated by a significant drop in pseudolikelihood, after which the scores stabilize as Hmax increases. This analysis suggests that Hmax = 1 provides the best estimate of the true number of reticulation events among the selected species.


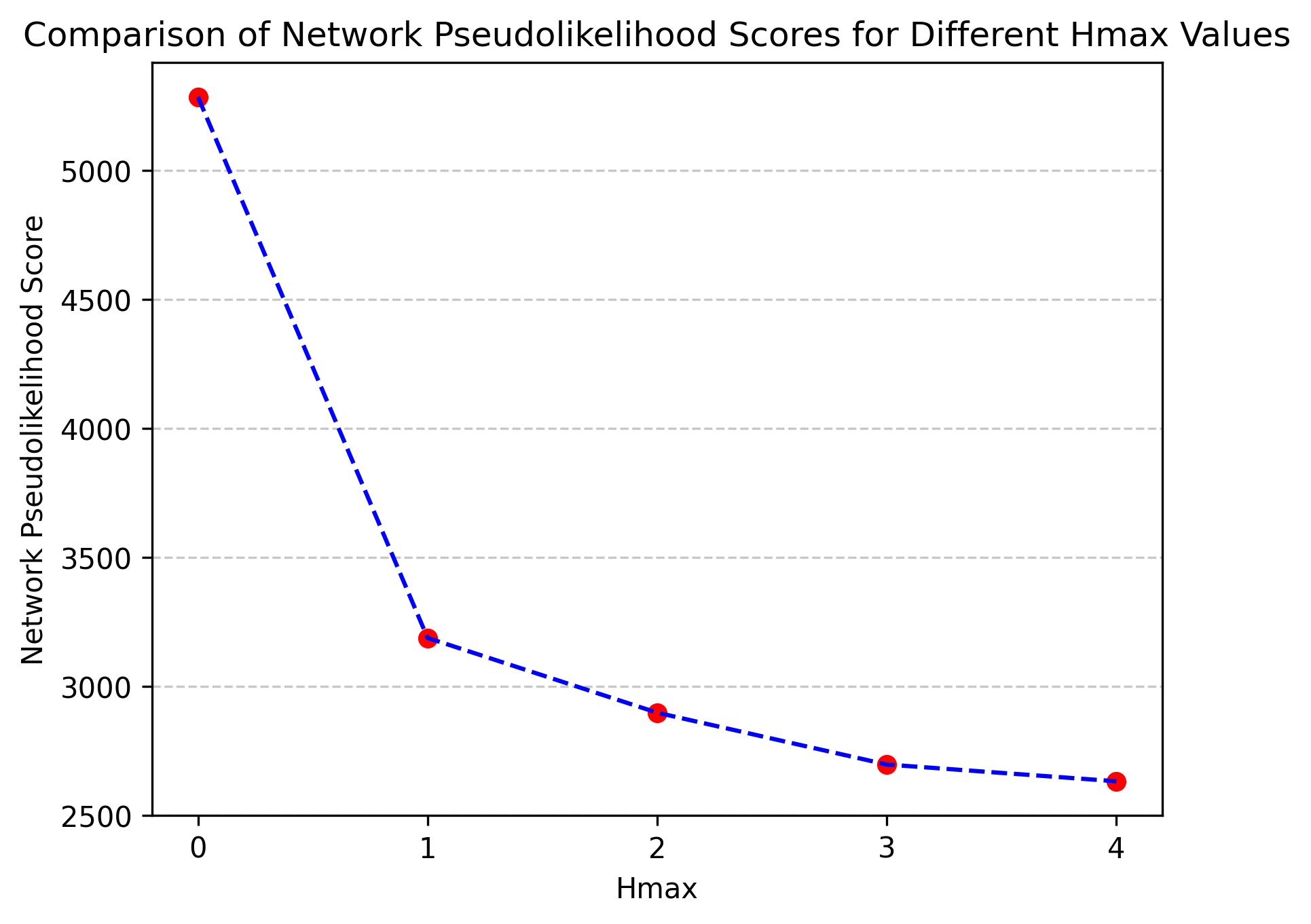

Supplement: Supplementary file 9 — Appendix S9. Comparison of pseudolikelihood scores for networks generated by SnaQ. [file AJB2-113-e70153-s004.docx]
