# Supplementary material for: Phylogenomics, ecomorphological evolution, and historical biogeography in Deuterocohnia (Bromeliaceae: Pitcairnioideae)
Source: Am J Bot. 2026 Jan 28;113(2):e70153. doi: 10.1002/ajb2.70153 (PMC12918849; doi:10.1002/ajb2.70153)
Supplement: Supplementary file 10 — Appendix S10. SnaQ results. [file AJB2-113-e70153-s010.docx]

**Appendix S10.** SnaQ results with runs = 20 and replicates = 30 with *H* (maximum number of reticulation) = 1 and 2. Gamma values, the proportional genomic contribution from parents, were reported. Trees were rooted with *Pitcairnia maidifolia* and *P. nuda.* Log-likelihoods for each networks are indicated at top of each network; the lower the likelihood, the more likely the network.
