# Supplementary material for: Phylogenomics, ecomorphological evolution, and historical biogeography in Deuterocohnia (Bromeliaceae: Pitcairnioideae)
Source: Am J Bot. 2026 Jan 28;113(2):e70153. doi: 10.1002/ajb2.70153 (PMC12918849; doi:10.1002/ajb2.70153)
Supplement: Supplementary file 12 — Appendix S12. Bayesian tree reconstructed from BEAST using plastid exon data set. [file AJB2-113-e70153-s001.docx]

**Appendix S12.** Bayesian tree reconstructed from BEAST using the plastid exon data set, with estimated ages and error bars representing the highest posterior density (HPD) interval at the 95% level. All nodes are highly supported with posterior probability of 1 except the nodes labelled with red support values.
