# Supplementary material for: Phylogenomics, ecomorphological evolution, and historical biogeography in Deuterocohnia (Bromeliaceae: Pitcairnioideae)
Source: Am J Bot. 2026 Jan 28;113(2):e70153. doi: 10.1002/ajb2.70153 (PMC12918849; doi:10.1002/ajb2.70153)
Supplement: Supplementary file 14 — Appendix S14. Most likely ancestral habitats at each node under DEC as implemented in BioGeoBears. [file AJB2-113-e70153-s011.docx]

**Appendix S14.** Most likely ancestral habitats at each node under DEC as implemented in BioGeoBears.
