# Supplementary material for: Phylogenomics, ecomorphological evolution, and historical biogeography in Deuterocohnia (Bromeliaceae: Pitcairnioideae)
Source: Am J Bot. 2026 Jan 28;113(2):e70153. doi: 10.1002/ajb2.70153 (PMC12918849; doi:10.1002/ajb2.70153)
Supplement: Supplementary file 15 — Appendix S15. Distribution of likely ancestral habitats at each node under DEC. [file AJB2-113-e70153-s015.docx]

**Appendix S15.** Distribution of likely ancestral habitats at each node under DEC. The light gray slice in the pie chart at certain nodes indicates the sum of likelihoods for all ancestral habitats with individual likelihoods <9.5%.
